# Postglacial Echoes: Parasite Genomics Uncover Environmental Changes in Postglacial European Lakes

**DOI:** 10.1111/mec.70039

**Published:** 2025-07-16

**Authors:** Mar Llaberia‐Robledillo, José Ignacio Lucas‐Lledó, Juan Antonio Balbuena, Jan Brabec, Julia Bilat, Rune Knudsen, Ole Seehausen, Isabel Blasco‐Costa

**Affiliations:** ^1^ Cavanilles Institute of Biodiversity and Evolutionary Biology University of Valencia Valencia Spain; ^2^ Department of Invertebrates Natural History Museum of Geneva Geneva Switzerland; ^3^ Institute of Parasitology, Biology Centre of the Czech Academy of Sciences České Budějovice Czech Republic; ^4^ Department of Arctic Biology The Arctic University of Norway Tromsø Norway; ^5^ Department of Fish Ecology and Evolution Centre of Ecology, Evolution and Biogeochemistry (CEEB), Eawag Swiss Federal Institute of Aquatic Science and Technology Kastanienbaum Switzerland; ^6^ Division of Aquatic Ecology & Evolution Institute of Ecology and Evolution, University of Bern Bern Switzerland

**Keywords:** ddRAD‐seq, demographic inference, freshwater, population genetics, Trematoda

## Abstract

Postglacial environmental changes have influenced biodiversity and species evolution, yet the genomic and demographic responses of parasites remain underexplored. This study investigates the population genetics and demographic history of the flatworm *Phyllodistomum umblae*, a generalist trematode at the definitive host level infecting *Coregonus* spp. across perialpine and subarctic postglacial lakes. Additionally, we compare its demographic patterns to *Proteocephalus fallax*, a whitefish specialist tapeworm, to elucidate how ecological strategies shape evolutionary responses to environmental fluctuations. Genomic data from ddRAD sequencing revealed clear genetic differentiation in *P. umblae* between subarctic and perialpine regions, likely driven by geographic isolation during glacial cycles. Low genetic differentiation suggests hydrological connectivity and the parasite's ability to utilise several host species as definitive hosts. Demographic inference uncovered distinct evolutionary trajectories between *P. umblae* and *Pr. fallax*. During the Last Glacial Period (~115–11 kya), *P. umblae* populations underwent declines, followed by rapid postglacial expansions after the Last Glacial Maximum (~15–10 kya). In contrast, *Pr. fallax* exhibited older historical fluctuations, including pronounced bottlenecks during the Middle Pleistocene (~300 kya). Its populations remained stable during the LGP, likely due to host persistence in glacial refugia unavailable in earlier glaciation periods. These findings align with the taxon pulse concept within the Stockholm Paradigm, highlighting how glacial cycles triggered episodic population contractions and expansions. By integrating genomic and historical data, this study (1) underscores parasites as models for understanding ecological and evolutionary processes and (2) provides insights into biodiversity resilience and adaptation to past and future environmental changes.

## 1 Introduction

Pleistocene glaciations, which occurred over the past 2.6 million years, were characterised by repeated cycles of glacial expansion and contraction that had a profound impact on the distribution and evolution of many organisms (Schluter 2000; Hewitt 2004; Schmitt 2007; Bernatchez et al. 2010). During glacial periods, species were often confined to refugia, that is, isolated areas where conditions remained favourable for survival (Willis and Whittaker 2000). These refugia served as reservoirs of genetic diversity, and during interglacial periods, the retreat of glaciers allowed species to expand into newly available habitats, triggering processes of colonisation and adaptation (Habel et al. 2010; Husemann et al. 2014). These glacial and postglacial dynamics align with the Stockholm Paradigm (Hoberg and Brooks 2015), a conceptual framework that links environmental change to patterns of diversification through four interacting processes: taxon pulse, ecological fitting, oscillation and geographic colonisation. Within this framework, the taxon pulse concept describes episodic expansions and contractions of species ranges in response to climatic fluctuations, leading to cycles of isolation, reconnection and adaptation (Erwin 1985; Agosta and Brooks 2020). This theoretical lens provides a valuable basis for interpreting how repeated glacial cycles may have shaped the evolutionary outcomes across species. Throughout the late Quaternary, significant environmental changes and dramatic climatic fluctuations profoundly impacted species distributions (Hewitt 2000; Smith et al. 2022). Thus, understanding how species evolved and adapted throughout these glacial and postglacial cycles is crucial for revealing broader patterns of biodiversity and evolution. Additionally, studying these past climatic shifts can provide critical information for anticipating future changes in the face of ongoing climatic change.

With the advent of novel DNA technologies, researchers can now uncover the genetic imprints of these historical shifts, offering powerful insights into the mechanisms underlying biodiversity and speciation (Cahill et al. 2013; Seehausen et al. 2014; Johannesson et al. 2020). Studies of the impact of Pleistocene glaciations have predominantly focused on free‐living organisms (e.g., Salvi et al. 2013; Cabanne et al. 2016; Nevado et al. 2018; Arcones et al. 2021), whereas the effect on parasites remains vastly underexplored. This lack of attention is problematic because parasite studies can potentially provide unprecedented insights not only into historical host geographical expansions and host–parasite interactions, but also into how ecosystems respond to dramatic climatic events. Parasites, due to their potentially higher mutation rates compared to their hosts, may accumulate genetic differences at a faster rate, which allows detecting demographic and evolutionary shifts with higher precision (Huyse et al. 2005). Thus, they can function as a ‘magnifying glass’, revealing subtle changes in host populations and environmental conditions that might not be easily detected focusing on the hosts themselves (Geraerts et al. 2022). Moreover, because parasite life cycles and dispersal are intricately tied to those of their hosts, parasites can provide valuable insights into the genetic structure of host populations, offering a broader understanding of ecological and evolutionary processes (Whiteman and Parker 2005; Gagne et al. 2022). Although previous studies have used parasites to infer host dynamics (Kmentová et al. 2019; Santoro et al. 2020), there has been limited focus on how parasites themselves respond to environmental changes. We propose that investigating the genomic diversity and population dynamics of parasites may provide key insights into their evolutionary trajectories, evaluate their resilience and identify their vulnerability to future environmental challenges, relevant in the current context of rapid global change.

Following the retreat of glaciers after the Last Glacial Maximum (LGM), ca.10,000–15,000 years ago, numerous post‐glacial lakes formed across Europe (Hewitt 2000; Brooks et al. 2011). The postglacial formation of these new environments created unique opportunities for studying evolutionary processes, serving as natural laboratories in which diversification occurred over shared timeframes (Schluter 1996; Hudson et al. 2011). The European whitefish ( *Coregonus lavaretus* species complex) has been extensively studied in this light (Østbye et al. 2005; Vonlanthen et al. 2012; Adams et al. 2016; Häkli et al. 2018; Selz et al. 2020; Crotti, Bean, et al. 2021; Crotti, Yohannes, et al. 2021). This species complex has undergone an intricate history of parallel diversification in lakes across the Northern Hemisphere, following the retreat of the ice shield after the LGM. Hence, whitefish exhibit an interesting evolutionary history across European freshwater systems (Østbye et al. 2005; Hudson et al. 2011) and are often used as a model system for studying rapid speciation and adaptive radiation (Hudson et al. 2007; Crotti, Bean, et al. 2021; Crotti, Yohannes, et al. 2021). However, the evolutionary trajectories of their parasitic fauna have been barely studied. Brabec et al. (2024) showed that the whitefish post‐glacial expansion and subsequent rapid diversification facilitated parasite colonisation and replicated spatial differentiation in these newly formed post‐glacial lakes. This whitefish diversification may have led to the creation of a variety of niches and ecological opportunities, providing parasites with diverse new resources to explore and exploit.

Here, we will focus on the population genomics of two parasitic platyhelminth species infecting European whitefish, *Phyllodistomum umblae* (Digenea) and *Proteocephalus fallax* (Eucestoda). The former is a generalist parasite at the definitive host level, infecting various salmoniform species of *Coregonus*, *Salmo*, *Salvelinus* and *Thymallus* (Moravec 2004; Soldánová et al. 2017; Faltýnková et al. 2020; Rochat et al. 2021). Although the life cycles of most *Phyllodistomum* species have not been fully elucidated, it is known that *P. umblae* successively infects freshwater bivalves (Sphaeriidae) as first intermediate hosts, and possibly Plecoptera nymphs as second intermediate hosts (Butorina et al. 2008; Petkevičiūtė et al. 2014, 2015; Siwertsson et al. 2016; Güven and Öztürk 2022) before reaching maturity in the definitive fish host. In contrast, *Pr. fallax* follows a more specific life cycle involving copepods (Cyclopidae and Diaptomidae) as intermediate hosts, with *Coregonus* spp. as its exclusive definitive host (Scholz 1999; Brabec et al. 2023; Scholz et al. 2024). The broader host range of *P. umblae* likely enhances its dispersal potential, as it can complete its life cycle in multiple fish species across different habitats. In contrast, the dependency of *Pr. fallax* on a single definitive host restricts its movement. Parasites infecting multiple definitive host species often show higher dispersal ability and reduced genetic structure (Huyse et al. 2005).

Brabec et al. (2024) explored the differentiation patterns of *Pr. fallax* across the same lakes covered in our study, showing that the external environment (i.e., abiotic environmental factors outside the host), host availability and microevolutionary forces, such as founder effects and genetic drift, shaped its evolution. These findings provide a valuable basis for comparing the differentiation patterns of *Pr. fallax* and *P. umblae* within the same postglacial ecosystems, which will be studied herein. The aim of this study is to elucidate the evolutionary dynamics underlying the demographic fluctuations of parasites in postglacial lakes and their different genetic signatures. To accomplish this, we used a double‐digestion restriction site‐associated DNA (ddRAD) sequencing protocol applied to *P. umblae* to obtain a high number of sufficiently variable molecular markers for a non‐model flatworm. In addition, we compare the demographic patterns between *P. umblae* and *Pr. fallax* based on previously published ddRAD data for the latter (Brabec et al. 2024). This enables a detailed investigation of population dynamics and demographic history for *P. umblae* and *Pr. fallax* across perialpine and subarctic postglacial lakes as independent replicates of colonisation events. We hypothesise that there will be low or no genetic differentiation within geographically proximate populations (i.e., within the same region) of the generalist flatworm *P. umblae*. The ability of the parasite to utilise diverse salmonid hosts would facilitate dispersal and gene flow across interconnected freshwater systems, hampering the emergence of significant local genetic structure. Additionally, and in line with the Pleistocene species range contractions and expansions (i.e., taxon pulses), we also hypothesise that lake populations of *P. umblae* and *Pr. fallax* have experienced founder events and population reductions followed up by demographic expansion driven by the availability and opportunity to exploit new hosts. *Phyllodistomum umblae* is expected to undergo rapid expansion post‐colonisation, whereas *Pr. fallax* would show a slower expansion due to its inability to utilise a broad host range for dispersion and establishment (Hoberg and Brooks 2008).

## 2 Methods

### 2.1 Data Acquisition, ddRAD Sequencing and Processing

**FIGURE 1:**
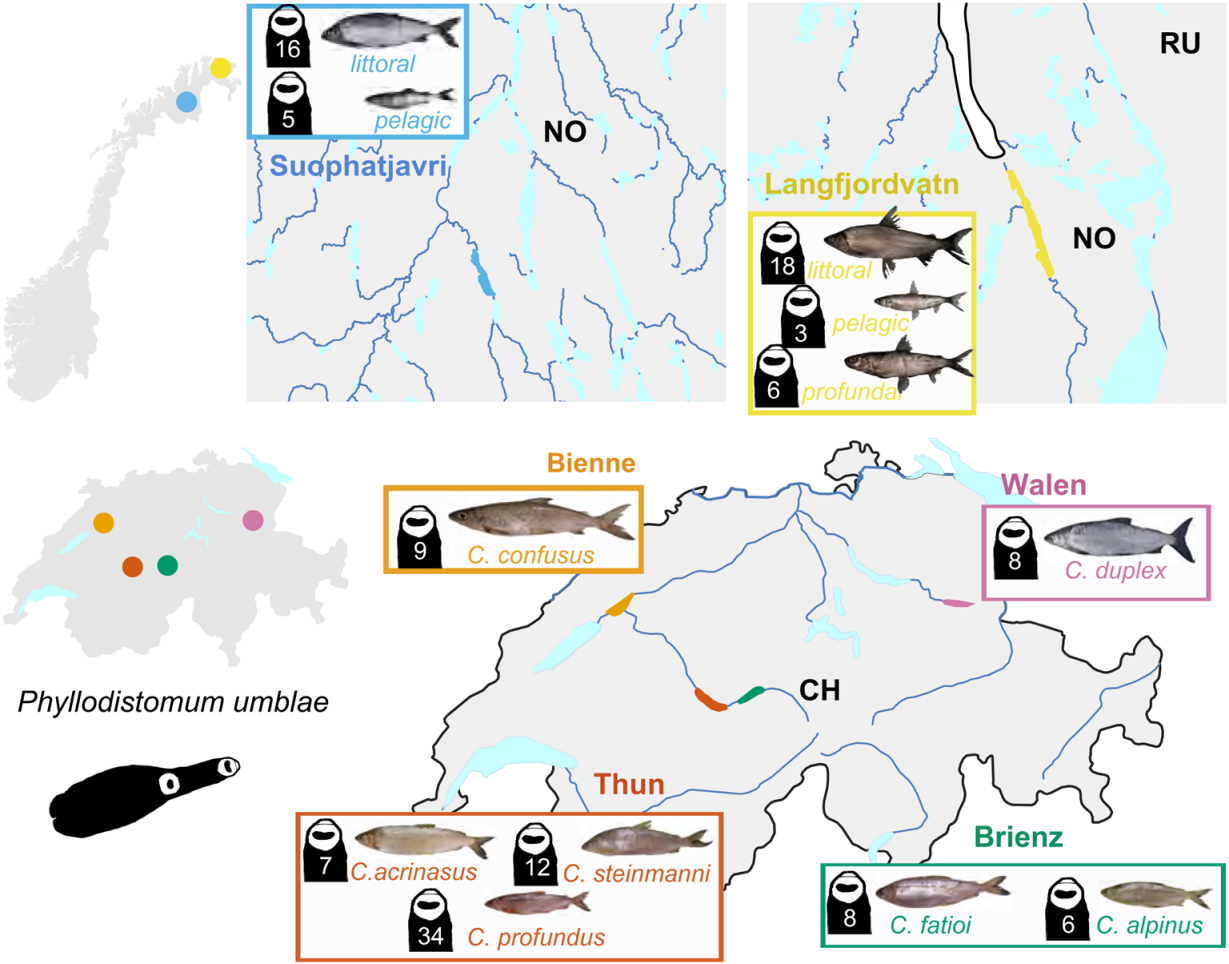
Sampling locations of *Phyllodistomum umblae* in the perialpine (four lakes in Switzerland ‐CH‐) and subarctic (two lakes in northern Norway ‐NO‐) regions (insets on the left). Lakes in each region are colour‐coded (enlarged maps: Suohpatjávri, blue; Langfjordvatn, yellow; Bienne, orange; Walen, pink; Thun, dark orange; Brienz, dark green). Illustrations of European whitefish species/ecotypes surveyed are accompanied by black silhouettes of *P. umblae* parasites, with the number of specimens genotyped from each host species indicated.

Specimens of *P. umblae* were collected from *Coregonus* spp. across four perialpine lakes in Switzerland (Lakes Bienne, Brienz, Thun and Walen) and two subarctic lakes in northern Norway (Suohpatjávri and Langfjordvatn), as described in Brabec et al. (2024) (Figure 1). Genomic DNA from 136 specimens of *P. umblae* was extracted and processed for ddRAD library construction, following the protocol outlined by Brabec et al. (2024). Around 60 ng gDNA per sample was doubly digested with frequent cutters NlaII and MseI, before ligating the adaptors, tagged with 24 different barcodes. We applied 20 cycles of PCR amplification with reverse primers tagged with 16 different indexes. Fragments between 320 and 500 bp were selected for sequencing in six lanes of Illumina HiSeq 2500 with 150 bp paired‐end reads. Demultiplexing and trimming of ddRAD reads were performed using IpyRAD's first step (Eaton and Overcast 2020), followed by quality assessment using FASTQC (Andrews 2010). High‐quality reads were then mapped to the reference genome (see next section) using bwa‐mem2 (Vasimuddin et al. 2019). Then, SAMtools (Li et al. 2009) was used to filter out unmapped reads and to retain reads with a mapping quality score above 20, followed by BAM compression and indexing. After filtering, we kept 131 samples of *P. umblae* to carry out the population genetic and demographic analysis. The initial dataset for *Pr. fallax* retrieved from Brabec et al. (2024) comprised 510 specimens. After filtering for specimens with conditions matching those of our study, we retained 447 samples for the demographic analysis.

### 2.2 Parasite Whole Genome Sequencing and Assembly

Conducting genomic analyses on small organisms, such as parasitic helminths, presents notable challenges because of the limited amount of tissue for the acquisition of sufficient high‐molecular‐weight genomic DNA (gDNA) and the risk of contamination with host tissues (Thorn et al. 2023). To address these constraints, we selected five extractions with the highest DNA quality and fragment size from fresh adult specimens collected from Arctic charr ( *Salvelinus alpinus* (Linnaeus, 1758)) from Skogsfjordvatn (69°56′30″ N, 19°09′22″ E, Norway) for sequencing with short‐read and long‐read methods. This combination was necessary to balance read length and accuracy: short‐read sequencing ensures high base accuracy, whereas long‐read sequencing enables better assembly of repetitive regions and provides longer contiguity (Nowak et al. 2019).

One specimen with 800 ng gDNA extract was used to construct an Illumina TruSeq DNA PCR‐free library with insert sizes of ~550 bp following the Illumina standard protocol and subjected to paired‐end (2 × 150 bp) sequencing on a NovaSeq6000 platform (Illumina). We used two additional specimen extractions with 400 ng gDNA each separately for library preparation and MinION long‐read sequencing (Oxford NanoporeTM Technologies), with GUPPY base calling using the High‐Accuracy models. Finally, two specimen extractions were combined to a total of 600 ng gDNA for SMRTbell library preparation and PacBio HiFi (Pacific Biosciences) sequencing on a Sequel II. Read quality was assessed using FASTQC (Andrews 2010) and genome size estimation was carried out on Illumina reads using a k‐mer depth frequency distribution analysis performed with GenomeScope (Vurture et al. 2017). We performed a hybrid genome assembly (Nowak et al. 2019) combining short and long‐reads methods, to generate a reference genome that is as complete as possible. PacBio raw reads were assembled, and a first polishing step followed, integrating MinION raw reads using FLYE v2.9.1 (Kolmogorov et al. 2019). We used Illumina reads to perform a second polishing step using ntHits and ntEdit (Warren et al. 2019). Finally, the completeness of the genome was assessed using BUSCO v5 (Manni et al. 2021) with the Metazoa database (metazoa_odb9).

### 2.3 Extraction of Genetic Markers and Haplotype Network

To investigate the genetic diversity and evolutionary relationships of *P. umblae*, we analysed both nuclear and mitochondrial genetic markers, which are widely recognised for their utility in evolutionary studies and specimen identification (Blasco‐Costa et al. 2016). Partial sequences of the nuclear large ribosomal subunit RNA gene (*lsrDNA*) and mitochondrial cytochrome c oxidase subunit 1 (*cox1*) were extracted from both the reference genome and ddRAD samples. We used phylogenetic tree reconstructions based on both genes to (i) verify the integrity of *P. umblae* as a single species, which was corroborated through molecular identification of specimens (Supporting Information S1), (ii) assess evolutionary relationships among *P. umblae* populations, and (iii) evaluate evolutionary relationships of the specimens from whitefish with those infecting other salmonids.

Demultiplexed and trimmed reads of the ddRAD samples were mapped either to the *cox1* or the *lsrDNA* reference sequences using bwa‐mem2 (Vasimuddin et al. 2019) and filtered using SAMtools (Li et al. 2009) (see details in Supporting Information S1). Consensus sequences corresponding to *cox1* or *lsrDNA* loci from all samples were aligned with MAFFT (Katoh et al. 2002), and sites with 75% or more missing data were removed using Gblocks (Castresana 2000). The *cox1* sequences, despite containing higher proportions of missing data (average of 23.5%, compared to 7.6% for *lsrDNA*), provided sufficient variation to allow further analyses. To examine haplotype relationships and assess genetic diversity across regions (Figure 1), we selected the *cox1* sequences to build the haplotype network using PEGAS (Paradis 2010) and geneHapR (Zhang et al. 2023) R packages (R Core Team 2024). Finally, the most dominant *cox1* haplotype (and the most complete sequence) from each lake, along with sequences of representative congeneric species of *Phyllodistomum* from GenBank, were used to generate a dataset for phylogenetic inference, focusing on evolutionary relationships within the genus (see Supporting Information S2).

### 2.4 Population Genetic Analyses

Since our ddRAD sequence coverage was low (3X–5X mean), we opted to work with ANGSD (Korneliussen et al. 2014), which is suitable to deal with low to medium sequencing depth data. This program operates within a probabilistic context using genotype likelihoods (i.e., probability of observing a particular sequence data given a particular genotype, at a particular location in the genome of a particular individual) (Korneliussen et al. 2014; Zhao et al. 2022), incorporating the uncertainty that may have been introduced in base calling, alignment, and assembly. We applied ANGSD to our ddRAD mapped reads to extract the genotype likelihoods, identify minor and major alleles, and call the SNPs. Only a subset of SNPs shared by at least 50% of the samples was retained. We used ngLSD (Fox et al. 2019) to estimate linkage disequilibrium among variable sites, and filtered them to keep r2 < 0.85. Genetic structure was investigated using PCAngsd (Meisner and Albrechtsen 2018), admixture proportions with NGSadmix (Skotte et al. 2013) for K values from 2 to 6 with up to 8000 iterations, and the most adequate number of clusters was evaluated with evalAdmix (Garcia‐Erill and Albrechtsen 2020). We estimated the identity by state (IBS) among samples and plotted it as a heatmap in R. We also estimated the site frequency spectrum (SFS) for each lake population and the fixation index (*F*_ST_) for every pair thereof with realSFS (Korneliussen et al. 2014).

### 2.5 Demographic History

Demographic changes in the last 700,000 years (beginning of the Middle Pleistocene) were investigated for *P. umblae* and *Pr. fallax*. Compressed fastq files for *Pr. fallax* sequences were retrieved from the data repository (NCBI's BioProject no. PRJNA910576, ddRAD accessions SAMN32132446–959, reference genome accession SAMN32134074) and were processed following the same pipeline as for *P. umblae*. Both species were sampled at the same time, locations, and came from the same host samples (Brabec et al. 2024). We used StairWay Plot 2 (Liu and Fu 2020) from the SFSs to infer demographic changes. We estimated the mutation rate following Lynch (2010), using a linear regression of log‐genome size and log‐mutation rate. This follows the assumption that mutation rates per nucleotide site per generation (u) scale with genome size (G). In addition to data provided by Lynch (2010), we included data from *Taenia* species (Wang et al. 2016) and from *Ligula intestinalis* (Nazarizadeh et al. 2023). The models yielded estimates of 3.66 × 10^−9^ and 5.06 × 10^−9^ substitutions/site/year for *P. umblae* and *Pr. fallax*, respectively. The contemporary effective population sizes and historical fluctuations were estimated for each lake population (six populations for both *P. umblae* and *Pr. fallax*). However, since the Stairway Plot relies on the informative power of the SFS (Terhorst and Song 2015; Reid and Pinsky 2022) the resolution and precision of the *P. umblae* dataset was insufficient to run the analysis at the lake level (see Supporting Information S5). Therefore, given the low *F*_ST_ values within one region (see results below and Brabec et al. 2024), we treated each region as a ‘metapopulation’ to achieve higher resolution in the demographic analysis.

## 3 Results

### 3.1 De Novo Genome Assembly

The draft genome of *P. umblae* had a total length of 450 Mb, comparable to the k‐mer estimate from Illumina raw reads (350 Mb). Sequencing data included 226,262 PacBio reads with a mean read length of 11,989 bp, 249,845 MinIon reads averaging 7248 bp, and 383,555,354 Illumina TrueSeq reads. BUSCO metrics indicated moderate genome completeness, with 44.6% of expected genes identified as complete (see Table S3), which is consistent with expectations for non‐model flatworms.

### 3.2 Haplotype Diversity and Differentiation of *Phyllodistomum umblae* Populations

**FIGURE 2:**
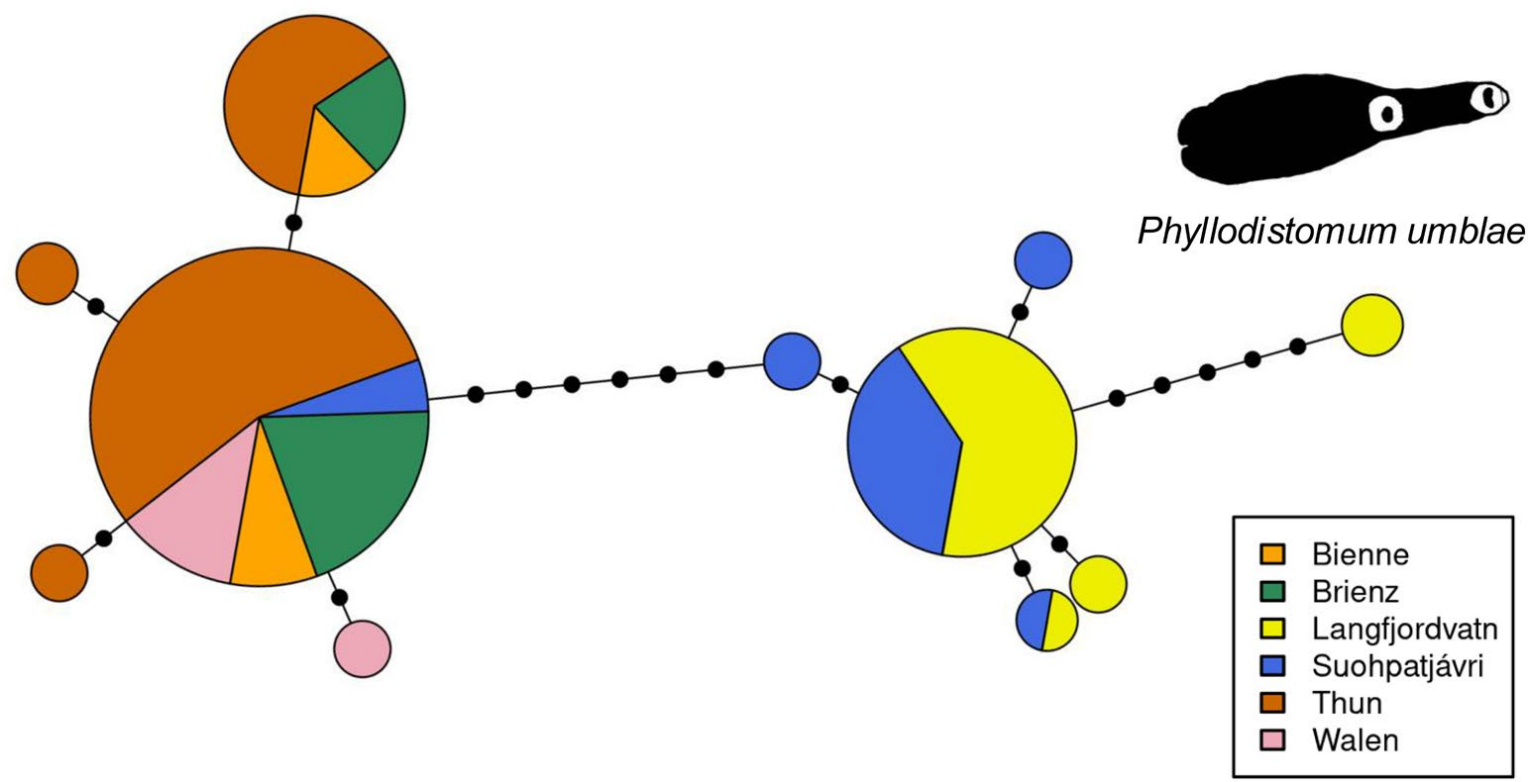
Haplotype network of *Phyllodistomum umblae cox1* gene, sampled from European whitefish (*Coregonus* spp.) hosts in perialpine (Bienne, Brienz, Thun and Walen) and subarctic (Langfjordvatn and Suohpatjávri) lakes. The network was computed from uncorrected distances among 131 sequences, 504 nucleotides long, with geneHapR (Zhang et al. 2023). Hash marks represent mutational steps and the size of each circle is proportional to the haplotype frequency.

The partial *cox1* and *lsrDNA* sequences extracted from the reference genomes were 1461 and 3873 bp long, respectively. The *cox1* and *lsrDNA* sequences extracted from all ddRAD samples contained varying proportions of missing data, with average completeness of *cox1* (23.5%) higher than that of *lsrDNA* (7.6%). The *lsrDNA* sequences from ddRAD samples were nearly identical across all samples (after removing sites with more than 5% missing data). The *cox1*‐based haplotype network revealed two distinct clusters of *P. umblae* separated by seven mutational steps (Figure 2). The first cluster corresponds to *P. umblae* populations from the subarctic region, whereas the second cluster includes populations from the perialpine region. Notably, the dominant haplotype in the perialpine cluster was also observed in three individuals from the subarctic Lake Suohpatjávri, highlighting a shared genetic variant between regions. Haplotypes within each cluster showed lower levels of differentiation, with few mutational steps separating them. Phylogenetic analyses depicted a strongly supported monophyletic clade, including representative sequences of *P. umblae* from whitefish from different lakes and conspecific sequences available from Genbank (Supporting Information S2).

### 3.3 Population Genetic Structure of *Phyllodistomum umblae*

**FIGURE 3:**
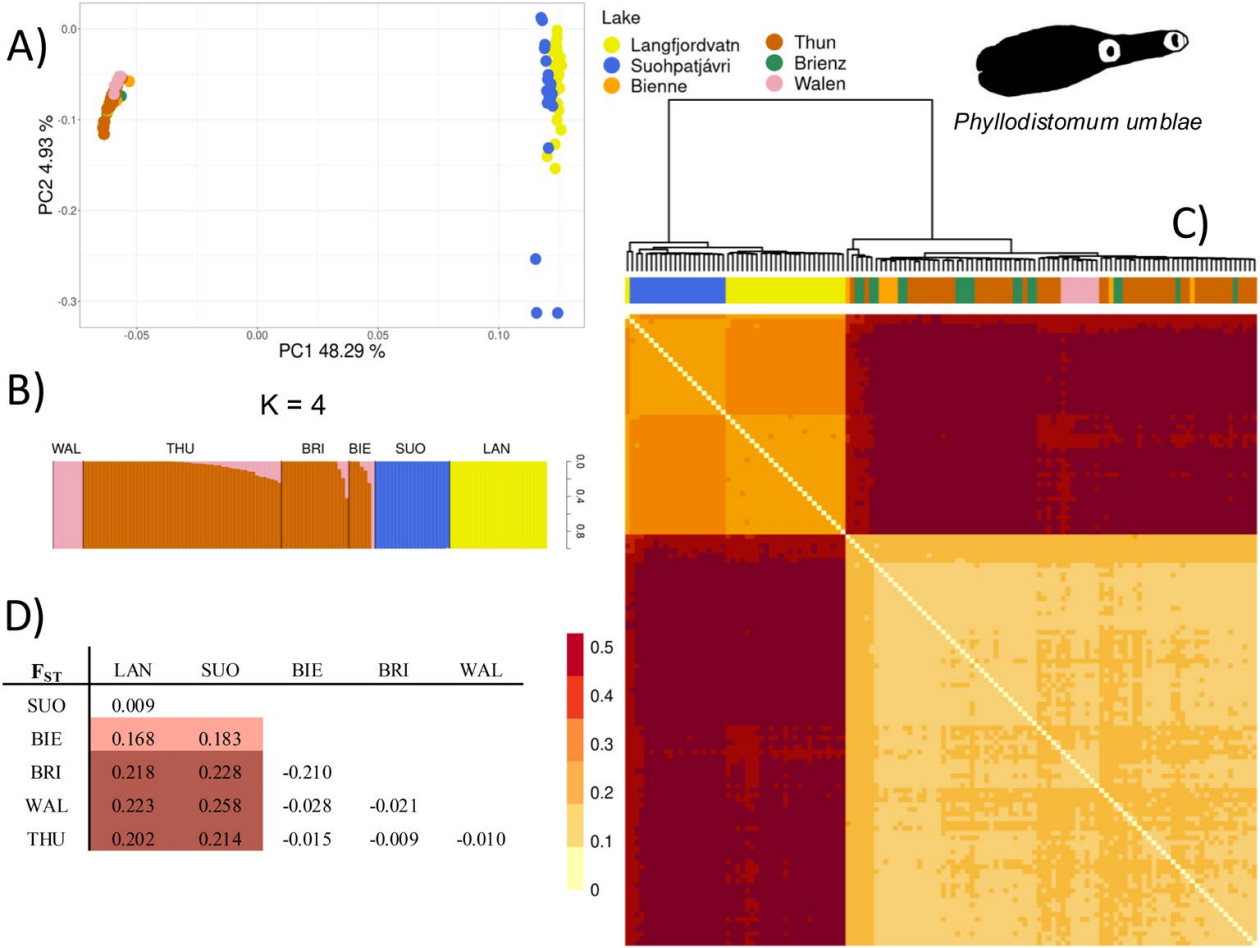
*Phyllodistomum umblae* population genetic structure across all lakes and regions. (A) Principal component analysis showing PC1 and PC2, with each dot representing an individual and colour representing the different lakes. Only the first axis differentiated the populations between the subarctic and perialpine regions. (B) Ancestry proportions from NGSadmix analysis, where the most adequate number of clusters for *P. umblae*, was *K* = 4, according to evalAdmix. (C) Heatmap representing the pairwise distances based on the identity by state (IBS) matrix estimated with ANGSD. Colours correspond to similarity between two multilocus genotypes, from yellow (low) to dark red (high). The populations of *P. umblae* are closely related within each region, but differentiated between regions. (D) Matrix of pairwise FST values between lakes, estimating genetic differentiation among P. umbale populations. Higher FST values (darker shades) indicates stronger genetic differentiation, mainly between the subarctic and perialpine regions, while populations within each region show lower differentaition.

The quality metrics showed that over 90% of the reads had average Phred scores of 30 or higher, indicating high‐quality sequences suitable for downstream analyses. The ddRAD sequencing of *P. umblae* produced a total of 238,613,554 high‐quality trimmed reads (average ± standard deviation of 1,754,511 ± 570,393 reads per individual). Mapping to the newly generated reference genome yielded an average alignment rate of 41.9% after data cleaning. Using ANGSD, we identified 36,056 bi‐allelic SNPs shared by at least 50% of the samples. The draft genome used as reference showed moderate completeness (BUSCO 44.6%), which may have limited the recovery of some genomic regions and contributed to the proportion of unmapped reads. The genetic structure of *P. umblae* populations revealed a clear differentiation between subarctic and perialpine regions, as shown by distinct clusters in the PCA (Figure 3A, PC1: 48.29%) and admixture plots (Figure 3B, *K* = 4). Subarctic populations from Langfjordvatn and Suohpatjávri were differentiated in the admixture analysis, with no evidence of mixed ancestry between the two lakes. In contrast, the perialpine populations (Bienne, Brienz, Thun and Walen) showed minimal differentiation in the PCA (Figure 3A) and in the IBS heatmap (Figure 3C). However, the Lake Walen population appeared distinct from the other perialpine lakes (Figure 3B, but also see Supporting Information S4). Pairwise *F*_ST_ values (Figure 3D) supported these findings, showing substantial differentiation between subarctic and perialpine regions (*F*_ST_ ~ 0.2) and none to minimal genetic differentiation across lakes within each region (*F*_ST_ ~ 0 to 0.1).

### 3.4 Demographic History

**FIGURE 4:**
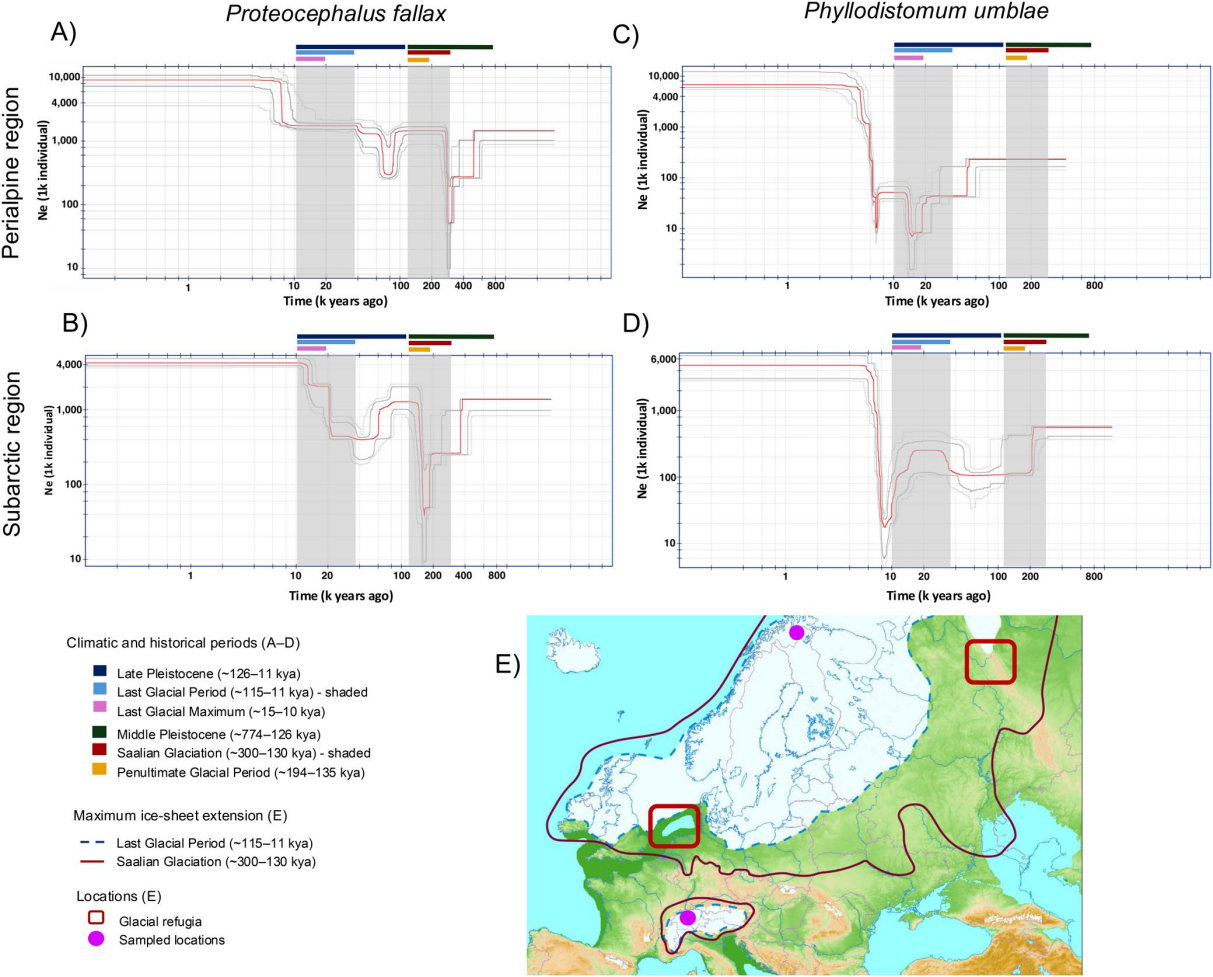
Demographic history inference of ancestral metapopulations: (A) perialpine region of *P. umblae*, (B) subarctic region of *P. umblae*, (C) perialpine region of *Pr. fallax*, and (D) subarctic region of *Pr. fallax*. The *X*‐axis represents time in thousands of years before present (kya) and the *Y*‐axis displays the mean effective population size (*N*_e_) in millions of individuals, both axis are on a logarithmic scale. StairWay plots based on site frequency spectrum (SFS). The red line in the plots represents the mean, grey lines indicate 75% (dark) and 95% (light) confidence intervals. (E) Map showing the extent of ice sheets during the Saalian Glaciation (~300–130 kya) (in dark red) and the Last Glacial Maximum (~15–10 kya) (in dark blue) (Ehlers et al. 2023; Held et al. 2024), with pink dots indicating the locations of the study areas, and red boxes the estimated position of glacial refugia (Østbye et al. 2005).

Demographic plots of *P. umblae* and *Pr. fallax* from the subarctic and perialpine metapopulations, spanning the Late Pleistocene (~126–11 kya) and Middle Pleistocene (~774–126 kya), revealed several demographic fluctuations (Figure 4). At the metapopulation level, the ranges of effective population size were 6–10 × 10^6^ and 4–10 × 10^6^ individuals for *P. umblae* and *Pr. fallax*, respectively. Historical fluctuations for *P. umblae* are inferred from the beginning of the Last Glacial Period (LGP; ~115–11 kya, see legend in Figure 4), whereas *Pr. fallax* exhibits evidence of earlier demographic events extending into the Middle Pleistocene. The demographic reconstructions are presented with confidence intervals: the dark grey line represents the 75% interval, and the light grey represents the 95%. These ranges illustrate the inherent uncertainty of the estimates, particularly regarding the timing of older demographic events, which should be interpreted with caution when using SFS‐based models such as Stairway Plot.

A significant bottleneck occurred in *Pr. fallax* populations ~200 to ~300 kya in ancestors of the subarctic and perialpine metapopulations respectively (Figure 4B,D), coinciding with the Saalian Glaciation in Europe (~300–130 kya), followed by a rapid recovery in population size. During the LGP, ancestors of both metapopulations underwent another significant decline in population size between ~60 and 80 kya. In the ancestors of the perialpine metapopulation, this decline was followed by a relatively rapid recovery, whereas in those of the subarctic metapopulation, the population size remained low and relatively stable for several thousand years, until the LGM, when the population size grew. The demographic history of *P. umblae* is marked by continuous population declines during the LGP (Figure 4A,C). In the ancestors of the subarctic metapopulation, an early decline occurred at the start of the LGP, followed by a stabilisation period, interrupted by a small population expansion ~30 kya, lasting until the LGM. Following the LGM, a pronounced bottleneck occurred ~8 kya, followed by rapid population recovery and expansion between ~8 and 6 kya, after which the population stabilised. Among the ancestors of the perialpine metapopulation, *P. umblae* experienced a continuous decline throughout the LGP, with a pronounced bottleneck ~15 kya, followed by a modest recovery. After the LGM, a second bottleneck between ~8 and 7 kya was observed, followed by rapid population growth over a relatively short period. Thus, both species show a pattern of repeated declines, genetic diversity loss, and partial recoveries, with population sizes eventually stabilising.

## 4 Discussion

The population dynamics and genetic patterns of parasitic species in postglacial lake environments remain relatively underexplored, leaving significant gaps in our understanding of how these organisms respond to environmental changes. Our results of several *P. umblae* populations reveal marked genetic differentiation between the subarctic and perialpine regions, whereas genetic structure across lakes within each region was low or absent. The latter result supports our initial hypothesis that proximate spatial distribution of lakes within regions would lead to local factors, such as shared host species (generalist parasite) and interconnected water systems, to favour genetic exchange within regions, thereby maintaining gene flow among lake populations. Demographic inference indicated that both *P. umblae* (this study) and *Pr. fallax* (data from Brabec et al. 2024) experienced a series of demographic fluctuations over an extended timeframe, spanning the Middle and Late Pleistocene (~126–11 kya). In both species, cycles of ice sheet expansion and contraction caused reductions in effective population size and genetic diversity, resulting in bottlenecks that left small founding populations. The subsequent recovery and expansion, that is, taxon pulse, of these populations support our hypothesis of postglacial expansion driven by recolonisation from isolated refugia populations. Thus, our findings underscore how recurring glacial environmental cycles have profoundly shaped the demographic trajectories of *P. umblae* and *Pr. fallax*. By examining these dynamics in parasite species, our study demonstrates how such organisms may respond to large‐scale climatic events, providing a novel way to study the ecological and evolutionary history of parasitic taxa and their free‐living host species.

*Phyllodistomum umblae* parasitizing *Coregonus* spp. was confirmed to represent a single species across regions, aligning with findings of limited genetic divergence among parasite populations infecting various fish species. The genetic differentiation observed in *P. umblae* between subarctic and perialpine regions conforms with the pattern of European whitefish recolonisation of Western Europe from two distinct refugia (Østbye et al. 2005). These recolonisation events created geographical segregation among whitefish populations (Hudson et al. 2007; Crotti, Bean, et al. 2021; Crotti, Yohannes, et al. 2021), and the combination of geographical distance between regions and restricted gene flow further contributed to the differentiation of their parasitic symbionts. However, despite their different recolonisation origin, genetic exchange between subarctic and alpine populations might have occurred. This idea is supported by two lines of evidence: (i) the mitochondrial haplotype shared between Suohpátjavri and perialpine populations of *P. umblae*, and (ii) the lower genetic differentiation observed in the perialpine population of Lake Bienne, located furthest north among the studied perialpine lakes. One plausible scenario is that populations originating from the southern glacial refugium (i.e., ancestors of perialpine populations) migrated northwards during postglacial recolonisation. This movement could have occurred through meltwater drainages, such as the Rhine and Elbe river systems, which provided connections to the Baltic Ice Sea and to Northern Europe as glaciers retreated (Østbye et al. 2005). Occasional long‐distance gene flow could also have occurred through meltwater drainage systems that connected northern and central European basins during deglaciation, or via anthropogenic fish translocations between lakes.

Although *P. umblae* is a generalist parasite that infects several salmonid hosts (Moravec 2004), the pattern of population differentiation within each region is quite different. In the subarctic region, Langfjordvatn and Suohpatjávri, located c. 280 km apart in separate water basins with no current hydrological connection, may have been influenced by larger freshwater systems formed during the glacial melting period, such as those associated with the Baltic Ice Lake (Ehlers 2022). Such past connections, followed by periods of isolation as the ice retreated and freshwater systems reorganised, could have contributed to *P. umblae* population differentiation through restricted migration and dispersal (i.e., vicariance), as for whitefish (Præbel et al. 2013). In contrast, the absence of substantial genetic structure of *P. umblae* in the perialpine region may result from the interconnected nature of the lakes within the Rhine watershed (Yanites et al. 2013). This watershed connects various lakes through river capture and incision events, creating a network of waterways that may facilitate (or have facilitated) gene flow among parasite populations. In fact, patterns of minor genetic differentiation of *P. umblae* within the perialpine region correlated with the varying strengths of interlake connections, suggesting that the degree of hydrological linkage influences gene flow dynamics. Additionally, this pattern is consistent with the population structure of the hosts, where weak or absent genetic differentiation has been reported among some perialpine Coregonus populations, particularly those inhabiting hydrologically connected systems (e.g., Vonlanthen et al. 2012; Selz et al. 2020; Crotti, Bean, et al. 2021; Crotti, Yohannes, et al. 2021). For example, Lakes Thun and Brienz were part of a larger postglacial lake as the ice retreated (Selz et al. 2020), which would explain the homogeneity in parasite populations observed. By contrast, *P. umblae* populations in Lake Walen exhibited slight genetic differentiation. This lake is hydrologically connected to Lake Zurich, but remains relatively isolated from Lakes Thun and Brienz and traces its origins to a larger postglacial lake system shaped by recolonisation events from multiple glacial refugia (Østbye et al. 2005; Hudson et al. 2007). Thus, the lake isolation from other perialpine lakes, combined with its colonisation history and anthropogenic activities (such as stocking practices), may account for the weak differentiation pattern observed here.

The regional differentiation in *P. umblae* aligns with patterns observed in *Pr. fallax* (Brabec et al. 2024). Similar to *P. umblae*, *Pr. fallax* exhibits regional genetic differentiation likely influenced by the geographic separation of *Coregonus* spp. host populations resulting from recolonisation from distinct refugia. However, *Pr. fallax* shows higher local genetic structuring, probably driven by its strict specificity to *Coregonus* spp. and other ecological traits, such as variations in habitat use and trophic behaviour (Brabec et al. 2024). This difference underscores that while both parasites exhibit regional genetic divergence influenced by geography, *Pr. fallax* appears to display an additional layer of local structuring driven by host ecological factors. Despite these patterns, neither parasite seems to have diverged to the extent of their *Coregonus* hosts. Although we surveyed 12 *Coregonus* species (Figure 1) across both regions, only a single genetic lineage of each parasite persists.

The demographic dynamics of *P. umblae* and *Pr. fallax* across the late Quaternary were revealed through the integration of parasitic genetic data and glacial historical factors. The observed low genetic differentiation among lakes within regions in both parasite species permitted the regions to be treated as single ‘metapopulations’. Then, the inferred demographic fluctuations of *P. fallax* extend back to ~400 kya, encompassing two full glacial cycles—the Penultimate Glacial Period (PGP, ~194–135 kya) and the LGP (~115–11 kya) ‐ whereas those of *P. umblae* date to ~200 kya, covering the LGP. This discrepancy in the temporal depth of inferred histories likely reflects both biological and technical factors. The larger number of specimens analysed for *Pr. fallax* (447 vs. 131 for *P. umblae*) and its higher sequencing depth likely improved the recovery of rare variants and the resolution of coalescent signals, enhancing the depth and accuracy of demographic inference. These factors may have contributed to the inference of a larger ancestral population size and allowed the demographic history of Pr. fallax to be traced further back in time (Terhorst and Song 2015; Lapierre et al. 2017). Furthermore, the use of RADseq and reliance on the SFS may have limited our ability to detect low‐frequency alleles, potentially reducing resolution for recent demographic events (Terhorst and Song 2015).

The decline in effective population size observed in *Pr. fallax* metapopulations during the Middle Pleistocene (~770–130 kya) coincides with the Saalian Glaciation (~300–130 kya) in northern Europe. This glaciation period encompassed three glacial cycles, characterised by repeated contractions and expansions of the ice sheets over thousands of years, with the PGP (~194–135 kya) marking the last of these cycles (Lauer and Weiss 2018). The timing of advancing ice sheets and harsh climatic conditions is congruent with the pronounced demographic decline in both ancestral metapopulations of *Pr. fallax* observed. Following the retreat of glaciers after the PGP, a rapid population expansion of *Pr. fallax* is evident, suggesting successful colonisation as the environment stabilised and freshwater resources became available. During the LGP, the Scandinavian ice sheet extended over northern Europe, reaching as far south as Germany and Poland, whereas during the Saalian Glaciation, it expanded even further south, covering parts of western Europe, including the Netherlands and northern France (Makkaveyev et al. 2022) (Figure 4). This differential glacial advance implies that some refugia available during the LGP were likely inaccessible during the Saalian Glaciation (Figure 4E). For instance, several LGP refugia in the British Isles were covered by ice during this period (Ehlers et al. 2023), which could explain the first pronounced population decline observed in the ancestors of the subarctic metapopulation of *Pr. fallax*. It is plausible that northern Fennoscandia was recolonised after the glaciation by fish populations expanding from eastern or southeastern refugia (see below) (Østbye et al. 2005), although it remains unclear whether this followed local extinction or colonisation of deglaciation areas. To date, no study has examined the demographic history of *Coregonus* populations in the subarctic. Our results therefore highlight the potential for future comparative studies integrating host and parasite genomic data to better understand parallel responses to past climate events.

During the LGP, *Pr. fallax*, which exclusively parasitises *Coregonus* species as definitive hosts—a cold‐water fish adapted to low temperatures (Elliott and Bell 2011) –, could sustain its life cycle within glacial refugia, enabling stable populations to persist throughout the glacial period. Similarly, the ancestors of the subarctic metapopulation of *P. umblae* experienced a period of stability during the LGP, likely due to the confinement in glacial refugia. This underscores the role of these refugia in promoting parasite population persistence and acting as reservoirs of genetic diversity during periods of extensive ice coverage (Hoberg et al. 2017). However, the most recent population decline post‐LGM in the ancestral perialpine *P. umblae* metapopulation is unexpected and suggests demographic contraction despite environmental stabilisation. Such fluctuations may result from genetic drift and selective pressures during colonisation (Blakeslee et al. 2020; Sromek et al. 2023), such as founder events and extinctions in the refugia and its proximity. In addition, the timing of the most recent population expansions in both parasite species suggests that the subarctic region, despite being farther north, was colonised earlier (~9–10 kya) than its perialpine counterpart (Figure 4). This could be due to the proximity of glacial refugia to the newly deglaciated areas (Østbye et al. 2005). Subarctic populations appear to have originated from the northeastern refugium west to the Ural mountains, which provided early access to deglaciated regions via freshwater corridors, allowing colonisation and population expansions ~9–10 kya. In contrast, perialpine populations possibly originated in southern refugia, located south of 53° N, which matches the maximum south‐ward extension of Saalian glaciation (Østbye et al. 2005). Despite the earlier ice sheet retreat in the perialpine region (~15 kya), the greater distance and geographical barriers could have delayed recolonisation, resulting in a later population expansion around ~8–7 kya.

After the LGM (~15–10 kya), *P. umblae*'s sharp population decline suggests founder events ~8 kya, followed by rapid population expansions likely driven by its ability to expand its host range and take advantage of available host opportunities (Hoberg and Brooks 2008; Araujo et al. 2015). In contrast, *Pr. fallax* populations, which depend on the survival and persistence of their exclusive definitive hosts, *Coregonus* spp., expanded more slowly (Brabec et al. 2024). These findings highlight how glacial cycles and environmental changes have differentially influenced the long‐term persistence, genetic structure, and evolutionary trajectories of parasite populations.

Parasites, despite being integral to ecosystem function (Brian 2023), remain understudied, particularly in how they respond to significant geological and climatic events. Our study addresses this gap, not only by illustrating how these organisms respond to past environmental changes, but by offering a framework for predicting future responses. Parasites may also serve as early indicators of ecosystem shifts, as their complex life cycles and sensitivity to host availability make them responsive to subtle environmental changes. In the Anthropocene, where rapid climate change and habitat loss are increasingly impacting ecosystems, such studies are essential, particularly for anticipating range shifts and the emergence of parasites (Kafle et al. 2020). The current loss of cold‐water habitats, essential for salmonid species and their parasites, underlines the urgency of understanding the cascading effects of climate change on these systems.

Overall, this work highlights the dual importance of parasites as both subjects of research and models for understanding broad‐scale ecological and evolutionary responses to environmental change. By integrating genetic data with historical climatic events, we revealed insights into the evolutionary history and vulnerability of parasite populations, providing a foundation for future conservation strategies and understanding biodiversity resilience in the face of global change.

## Author Contributions

I.B.‐C., J.A.B., M.L.‐R. and J.I.L.‐L. conceived the study; I.B.‐C., R.K., J.B.r, O.S. and J.B. contributed to data collection. M.L.‐R. performed the analyses. M.L.‐R. led the writing, and I.B.‐C., J.A.B. and J.I.L.‐L. contributed to the writing of the manuscript. All authors contributed critically to the drafts and gave final approval for publication.

## Disclosure

Data and Code Accessibility: All collapsed and paired‐end sequence data for samples sequenced in this study are available in compressed fastq format through NCBI's BioProject no. PRJNA1282119, together with rescaled and trimmed bam sequence alignments against the reference genome. All scripts used to perform the analyses presented in this paper are available through Zenodo and GitHub (10.5281/zenodo.15776244).

## Conflicts of Interest

The authors declare no conflicts of interest.

## Supporting information


**Figure S1.** Phylogenetic interrelationships of *Phyllodistomum* species parasitizing salmonids and the position of *Phyllodistomum umblae* within the group.
**Figure S2.** Phylogenetic interrelationships of *Phyllodistomum* species parasitizing salmonids and the position of *Phyllodistomum umblae* within the group.
**Figure S3.** Principal component analysis across perialpine lakes for *Phyllodistomum umblae*.
**Figure S4.** Principal component analysis across subarctic lakes for *Phyllodistomum umblae*.
**Figure S5.** Ancestry proportions from NGSadmix analysis, where the most adequate number of clusters for perialpine populations of *P. umblae* was *K* = 4 according to evalAdmix (residuals closest to 0).
**Figure S6.** Ancestry proportions from NGSadmix analysis, where the most adequate number of clusters for subarctic populations of *P. umblae* was *K* = 2 or *K* = 3 (both with the same probability) according to evalAdmix.
**Figure S7.** Demographic inference for each population (i.e., lake) in perialpine and subarctic region of *Pr. fallax*.
**Figure S8.** Estimation of base substitution rate per nucleotide site per generation with genome size.
**Table S1.** *Phyllodistomum* species accession information for *lsrDNA* gene, partial sequences retrived from Genbank.
**Table S2.** *Phyllodistomum* species accessions information for *cox1* gene, partial sequences retrived from GenBank.
**Table S3.** Summary statistics of genetic diversity in *Phyllodistomum umblae* populations from each lake.
**Table S4.** Summary statistics and BUSCO results for the de‐novo genome assembly for *Phyllodistomum umblae*.
**Table S5.** Comparisons of *Phyllodistomum umblae* population genetic structure (*F*_ST_) among lakes.

## Data Availability

All clean data and the scripts used to perform the analyses presented in this manuscript are available through the following Zenodo DOI: 10.5281/zenodo.15776244. See Data Accessibility for raw data.
