# Supplementary material for: Postglacial Echoes: Parasite Genomics Uncover Environmental Changes in Postglacial European Lakes
Source: Mol Ecol. 2025 Jul 16;34(17):e70039. doi: 10.1111/mec.70039 (PMC12376957; doi:10.1111/mec.70039)
Supplement: Supplementary file 1 — Figure S1. Phylogenetic interrelationships of Phyllodistomum species parasitizing salmonids and the position of Phyllodistomum umblae within the group. Figure S2. Phylogenetic interrelationships of Phyllodistomum species parasitizing salmonids and the position of Phyllodistomum umblae within the group. Figure S3. Principal component analysis across perialpine lakes for Phyllodistomum umblae. Figure S4. Principal component analysis across subarctic lakes for Phyllodistomum umblae. Figure S5. Ancestry proportions from NGSadmix analysis, where the most adequate number of clusters for perialpine populations of P. umblae was K = 4 according to evalAdmix (residuals closest to 0). Figure S6. Ancestry proportions from NGSadmix analysis, where the most adequate number of clusters for subarctic populations of P. umblae was K = 2 or K = 3 (both with the same probability) according to evalAdmix. Figure S7. Demographic inference for each population (i.e., lake) in perialpine and subarctic region of Pr. fallax. Figure S8. Estimation of base substitution rate per nucleotide site per generation with genome size. Table S1. Phyllodistomum species accession information for lsrDNA gene, partial sequences retrived from Genbank. Table S2. Phyllodistomum species accessions information for cox1 gene, partial sequences retrived from GenBank. Table S3. Summary statistics of genetic diversity in Phyllodistomum umblae populations from each lake. Table S4. Summary statistics and BUSCO results for the de‐novo genome assembly for Phyllodistomum umblae. Table S5. Comparisons of Phyllodistomum umblae population genetic structure (F ST) among lakes. [file MEC-34-e70039-s001.docx]

SUPPLEMENTARY MATERIAL FOR: **Postglacial echoes: Parasite genomics uncover environmental changes in postglacial European lakes**

Mar Llaberia-Robledillo^1,2*^, José Ignacio O^1^, Juan Antonio Balbuena^1^, Jan Brabec^2,3^, Rune Knudsen^4^, Ole Seehausen^5,6^, Isabel Blasco-Costa^2^

^1^ Cavanilles Institute of Biodiversity and Evolutionary Biology, University of Valencia, Valencia, Spain

^2^ Department of Invertebrates, Natural History Museum of Geneva, Geneva, Switzerland

^3^ Institute of Parasitology, Biology Centre of the Czech Academy of Sciences, České Budějovice, Czech Republic

^4^ Department of Arctic Biology, The Arctic University of Norway, Tromsø, Norway

^5^ Department of Fish Ecology and Evolution, Centre of Ecology, Evolution and Biogeochemistry (CEEB), Eawag Swiss Federal Institute of Aquatic Science and Technology, Seestrasse 79, CH-6047 Kastanienbaum, Switzerland.

^6^ Division of Aquatic Ecology & Evolution, Institute of Ecology and Evolution, University of Bern, Baltzerstr. 6, CH-3012 Bern, Switzerland.

* Corresponding author: [mar.llaberia@uv.es](mailto:mar.llaberia@uv.es)

SECTIONS

[S1. BIOINFORMATIC PIPELINES 2](#_Toc190165625)

[EXTRACTION AND PROCESSING OF GENETIC MARKERS 2](#_Toc190165626)

[IDENTIFICATION OF SPECIMENS 2](#_Toc190165627)

[S2. PHYLOGNETIC TREES RECONSTRUCTIONS 3](#_Toc190165628)

[PHYLOGENETIC TREE AT THE GENERIC LEVEL 3](#_Toc190165629)

[ACCESSIONS DETAILS 6](#_Toc190165630)

[S3. GENERAL STATISTICS 8](#_Toc190165631)

[GENETIC DIVERSITY 8](#_Toc190165632)

[REFERENCE GENOME STATISTICS 8](#_Toc190165633)

[S4. EXTENDED POPULATION GENETIC ANALYSIS 9](#_Toc190165634)

[PCA BY REGION 9](#_Toc190165635)

[ADMIXTURE BY REGION 10](#_Toc190165636)

[COMPARISON F_ST_ BETWEEN POPULATIONS 12](#_Toc190165637)

[S5. EXTENDED DEMOGRAPHIC INFERENCE ANALYSIS 13](#_Toc190165638)

[DEMOGRAPHIC INFERENCE BY LAKE POPULATIONS 13](#_Toc190165639)

[ESTIMATION OF EVOLUTIONARY RATE 14](#_Toc190165640)

[REFERENCES 16](#_Toc190165641)

# S1. BIOINFORMATIC PIPELINES

All the pipelines and scripts used for this study are in Zenodo and GitHub (10.5281/zenodo.15776244)

## EXTRACTION AND PROCESSING OF GENETIC MARKERS

To investigate the genetic diversity and evolutionary relationships of *Phyllodistomum umblae,* we extracted two widely used genetic markers, the mitochondrial cytochrome c oxidase subunit 1 (*cox1*) and the nuclear large subunit ribosomal DNA (*lsrDNA*), from both the reference genome and all individual samples.

***Extraction from the Reference Genome***

To identify the contigs containing these markers, we retrieved reference sequences of *cox1* and *lsrDNA* from *P. umblae* available in GenBank (Accession number for *lsrDNA*: MT076068). However, no cox1 sequence for P. umblae is available in GenBank. Therefore, we created a database using makeblastdb with all sequences from the same genus or closely related to *P. umblae*. We then mapped them to the reference genome and extracted the contig with the highest similarity, corresponding to the cox1 gene of *P. umblae*. For *lsrDNA* sequence from Genbank was aligned to the de novo *P. umblae* reference genome with blastn (Camacho et al., 2009) on a Linux Ubuntu environment to locate the most similar sequences. Then we used ape (Paradis & Schliep, 2019) in R (R Core Team, 2024) to extract the corresponding genomic regions.

***Extraction from Individual Samples***

For each *P. umblae* specimen, we mapped demultiplexed and quality-trimmed reads to the *cox1* and *lsrDNA* reference sequence extracted from the reference genome (detailed above) using bwa-mem2 (Vasimuddin et al., 2019). Mapped reads were then processed with samtools (Li et al., 2009) to extract consensus sequences for each sample, generating raw alignments for both markers across all individuals. These alignments were subsequently filtered to remove low-quality sequences and sites with excessive missing data (see filtering details below).

## IDENTIFICATION OF SPECIMENS

To confirm the species identity of *P. umblae* in our samples, we conducted both genetic and morphological identification approaches. Morphological identification of specimens was conducted under a stereomicroscope, previous to processing the specimens for DNA extraction. Then, we used the two genetic markers extracted from our samples, *cox1* and *lsrDNA*, to confirm species identification through sequence comparison with publicly available data. Each consensus sequence obtained from our dataset was queried against the NCBI GenBank database using BLAST (Altschul et al., 1990) to assess sequence similarity with previously deposited *Phyllodistomum* species. Species-level identification was determined based on a preliminary phylogenetic analysis (see details below) including all generated sequences and closely related congeneric species. This allowed us to validate the taxonomic assignment of our specimens and confirm their correspondence with *P. umblae*.

# S2. PHYLOGNETIC TREES RECONSTRUCTIONS

## PHYLOGENETIC TREE AT THE GENERIC LEVEL

Phylogenetic relationships within *Phyllodistomum* were reconstructed based on the most common haplotypes identified in each lake, as determined by the haplotype network presented in Figure 2 of the manuscript, hereafter referred to as sequences of *P. umblae* representatives. To expand the analysis, sequences from *Phyllodistomum* species and outgroup taxa were retrieved from genomic repositories such as NCBI and ENA (detailed below). A comprehensive dataset was compiled for both *cox1* and *lsrDNA* genes, and aligned using the translational L-INS-i algorithm in MAFFT (Katoh et al., 2002). The alignments underwent trimming and filtering to ensure data quality; conservative blocks were retained using Gblocks (Castresana, 2000), requiring at least 85% sequence agreement across the alignment. Sequences containing more than 75% missing data were excluded from further analyses.

Phylogenetic analyses were performed under the maximum likelihood (ML) criterion using RAxML-Next Generation (Kozlov et al., 2019). Nodal support was evaluated through 1,000 non-parametric bootstrap replicates, employing Felsenstein's Bootstrap Proportion (FBP). The best-fitting model of nucleotide evolution for both *cox1* and *lsrDNA* genes was identifed as GTR + G, based on the corrected Akaike Information Criterion (AIC) with RAxML-NG.

For the selected *P. umblae* representatives, missing data ranged from 9.9% to 29.7% for *cox1* and from 1.4% to 7.7% for *lsrDNA*. ML analyses based on both genes generated consistent and well-supported phylogenetic trees. In the *cox1* tree (Fig. S1), *P. umblae* samples formed a monophyletic clade (BP = 98), distinct from other *Phyllodistomum* species. Representatives from different lakes, such as Langfjordvatn and Suohpatjávri, exhibited closer relationships within the clade. Similarly, the *lsrDNA* reconstruction (Fig. S2) revealed a robust *P. umblae* clade (BP = 98), incorporating both our samples and sequences of *P. umblae* retrieved from GenBank into a cohesive group.


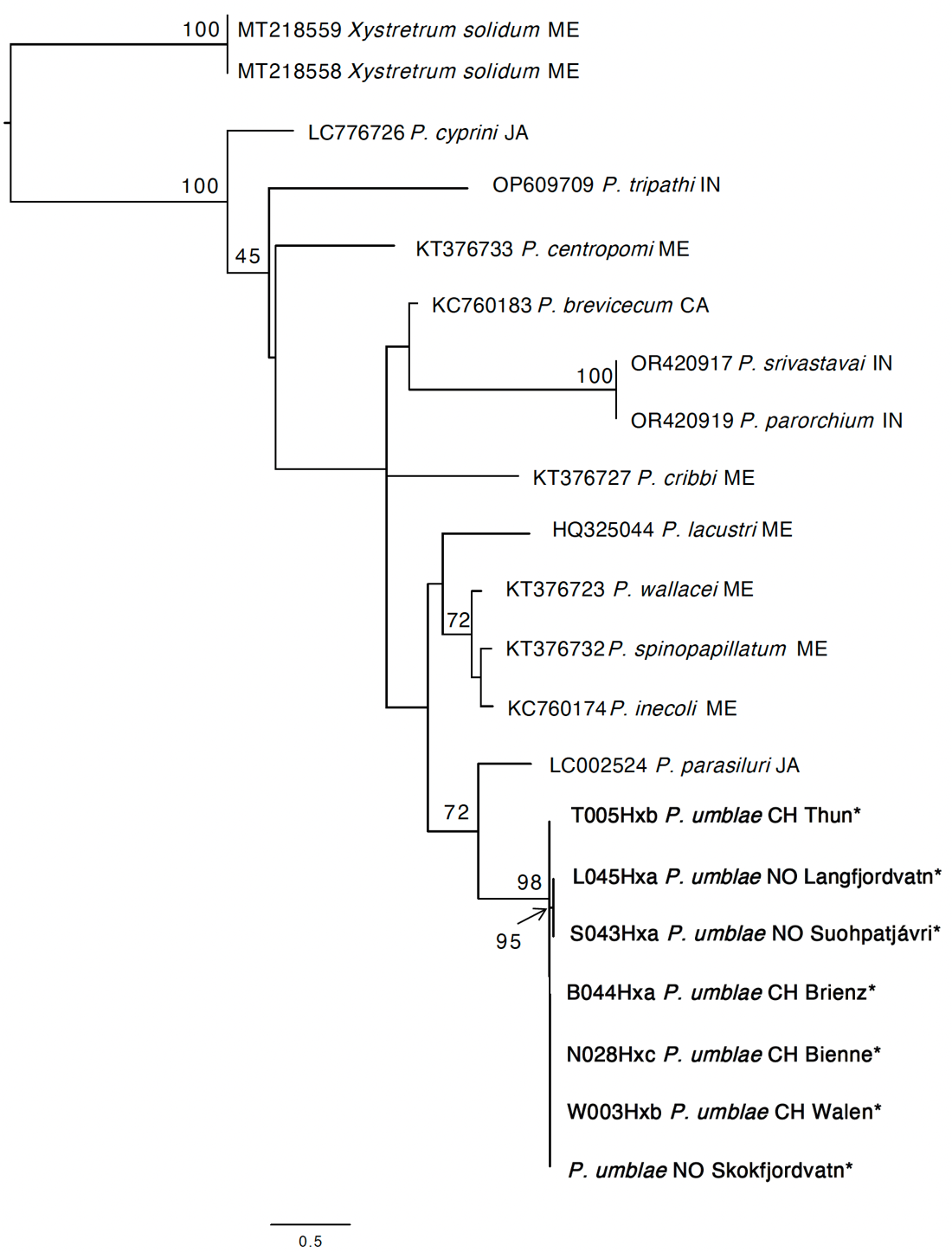


Fig S1. Phylogenetic interrelationships of *Phyllodistomum* species parasitizing salmonids and the position of *Phyllodistomum umblae* within the group. Maximum likelihood estimate based on *cox1* data analyzed as a single partition. The representative specimens marked with asterisks and in bold were obtained in the present study. Branch length scale bar represents the number of substitutions per site. Nodal values show non-parametric standard bootstrap support (1,000 replicates) above 70.


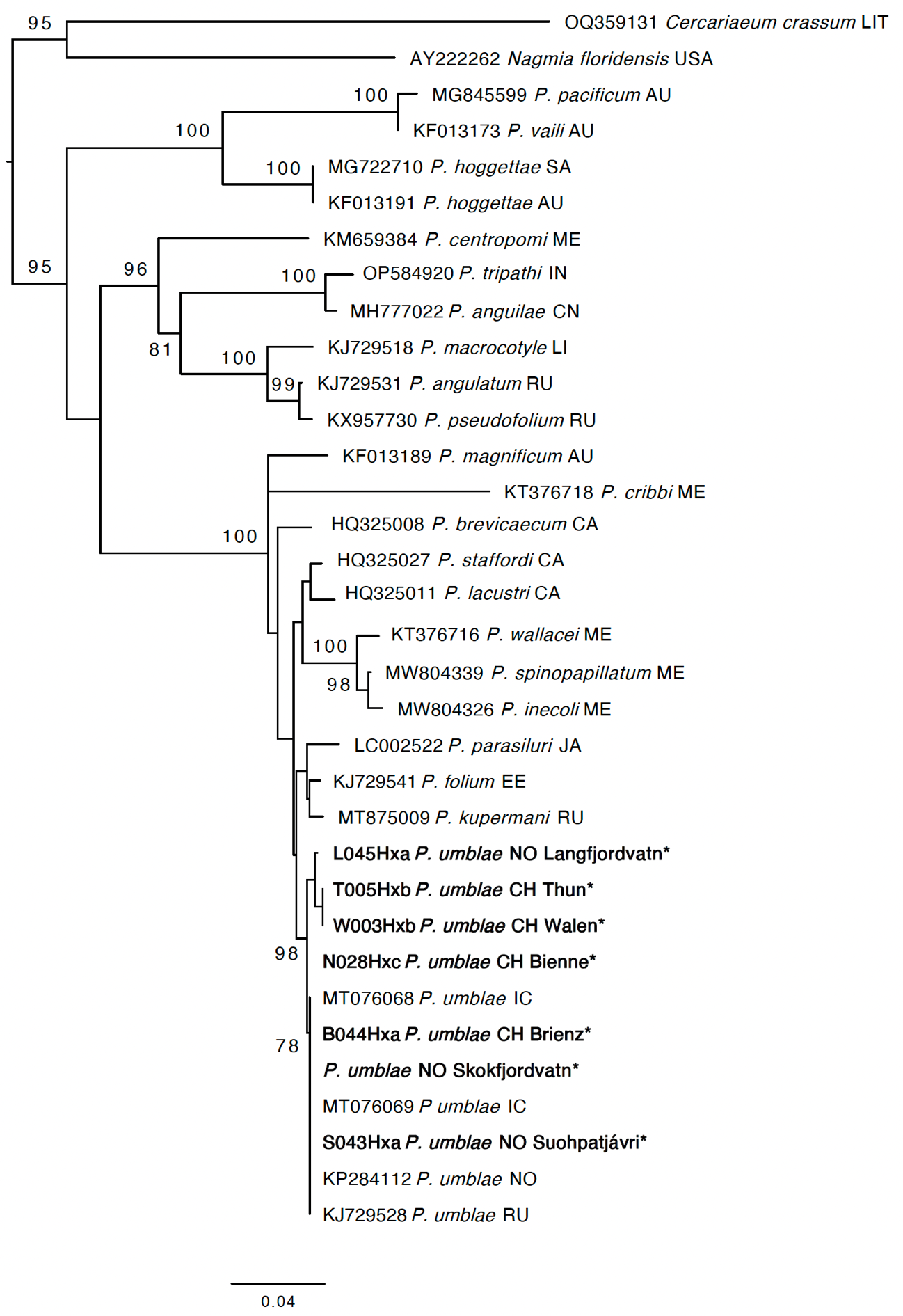


Fig S2. Phylogenetic interrelationships of *Phyllodistomum* species parasitizing salmonids and the position of *Phyllodistomum umblae* within the group. Maximum likelihood estimate based on *lsrDNA* data analyzed as a single partition. The representative specimens marked with asterisks and in bold were obtained in the present study. Branch length scale bar represents the number of substitutions per site. Nodal values show non-parametric standard bootstrap support (1000 replicates) above 70.

## ACCESSIONS DETAILS

To ensure transparency and reproducibility of the phylogenetic analyses, we provide the information on the sequences of *Phyllodistomum* species extracted from GenBank, which were used for constructing the different individual and genus-level phylogenetic trees shown in this section.

Table S1. *Phyllodistomum* species accession information for *lsrDNA* gene, partial sequences retrived from Genbank.

| **Accession** | **Scientific name** | **Host** | **Country** | **Reference** |
| --- | --- | --- | --- | --- |
| MH777022 | *P. anguilae* | *Siniperca scherzeri* | China | Zhang et al., unpublished |
| KJ729531 | *P. angulatum* | *Sander lucioperca* | Russia | Stunžėnas et al., 2017 |
| HQ325008 | *P. brevicecum* | *Umbra limi* | Canada | Rosas-Valdez et al., 2011 |
| KM659384 | *P. centropomi* | *Centropomus parallelus* | Mexico | León et al., 2015 |
| KT376718 | *P. cribbi* | *Zoogoneticus quitzeoensis* | Mexico | León et al., 2015 Zootaxa |
| KJ729541 | *P. folium* | *Pisidium amnicum* | Estonia | Petkevičiūtė, Stunžėnas, et al., 2015 |
| MG722710 | *P. hoggettae* | *Plectropomus areolatus* | Saudi Arabia | Al-Ghamdi et al., 2021 |
| KF013191 | *P. hoggettae* | *Plectropomus leopardus* | Australia | Cutmore et al., 2013 |
| MW804326 | *P. inecoli* | *Profundulus oaxacae* | Mexico | Pinacho-Pinacho et al., 2021 |
| MT875009 | *P. kupermani* | *Perca fluviatilis* | Russia | Petkevičiūtė et al., 2020 |
| HQ325011 | *P. lacustri* | *Noturus flavus* | Canada | Rosas-Valdez et al., 2011 |
| KJ729518 | *P. macrocotyle* | *Dreissena polymorpha* | Lithuania | Petkevičiūtė, Stunžėnas, et al., 2015 |
| KF013189 | *P. magnificum* | *Tandanus tandanus* | Australia | Cutmore et al., 2013 |
| MG845599 | *P. pacificum* | *Pantolabus radiatus* | Australia | Cutmore & Cribb, 2018 |
| LC002522 | *P. parasiluri* | *Silurus asotus* | Japan | Urabe et al., 2015 |
| KX957730 | *P. pseudofolium* | *Pisidium amnicum* | Russia | Stunžėnas et al., 2017 |
| MW804339 | *P. spinopapillatum* | *Profundulus oaxacae* | Mexico | Pinacho-Pinacho et al., 2021 |
| HQ325027 | *P. staffordi* | *Ameiurus melas* | Canada | Rosas-Valdez et al., 2011 |
| OP584920 | *P. tripathi* | *Xenentodon cancila* | India | Rajput et al., unpublished |
| KP284112 | *P. umblae* | *Pisidium hibernicum* | Norway | Petkevičiūtė, Kudlai, et al., 2015 |
| KJ729528 |  | *Coregonus albula* | Russia | Petkevičiūtė, Stunžėnas, et al., 2015 |
| MT076068 |  | *Salvelinus alpinus* | Iceland | Faltýnková et al., 2020 |
| MT076069 |  |  |  |  |
| KF013173 | *P. vaili* | *Mulloidichthys flavolineatus* | Australia | Cutmore et al., 2013 |
| KT376716 | *P. wallacei* | *Xenotaenia resolanae* | Mexico | León et al., 2015 Zootaxa |
| OQ359131 | *Cercariaeum crassum* | *Rutilus rutilus* | Japan | Petkevičiūtė et al., 2023 |
| AY222262 | *Nagnua floridensis* | *Rhinopterea bonasus* | USA | Olson et al., 2003 |
| De-novo  Assembly* | *P. umblae* | *Salvelinus alpinus* | Norway | Present study |
| L049Hxa* |  | *Coregonus lavaretus* |  |  |
| S043Hxa* |  | *Coregonus lavaretus* |  |  |
| N092Hxb* |  | *Coregonus confusus* | Switzerland |  |
| T005Hxb* |  | *Coregonus steinmanni* |  |  |
| W006Hxb* |  | *Coregonus dúplex* |  |  |
| B110Hxa* |  | *Coregonus fatioi* |  |  |

* **GenBank accession numbers: draft genome: JBPKAA000000000, partial sequences: PV833202-PV833207**

Table S2. *Phyllodistomum* species accessions information for *cox1* gene, partial sequences retrived from GenBank.

| **Accession** | **Scientific name** | **Host** | **Country** | **Reference** |
| --- | --- | --- | --- | --- |
| KT376733 | *P. centropomi* | *Centropomus parallelus* | Mexico | León et al., 2015 |
| KT376732 | *P. spinopapillatum* | *Profundulus balsanus* |  |  |
| KT376723 | *P. wallacei* | *Xenotaenia resolanae* |  |  |
| KT376727 | *P. cribbi* | *Zoogoneticus quitzeoensis* |  |  |
| LC776726 | *P. cyprini* | *Cyprinus carpio* | Japan | Gosho et al., 2023 |
| KC760183 | *P. brevicecum* | *Umbra limi* | Canada | Razo-Mendivil et al., 2013 |
| KC760174 | *P. inecoli* | *Heterandria bimaculata* | Mexico |  |
| HQ325044 | *P. lacustri* | *Ictalurus pricei* | Canada | Rosas-Valdez et al., 2011 |
| LC002524 | *P. parasiluri* | *Silurus asotus* | Japan | Urabe et al., 2015 |
| OR420919 | *P. parorchium* | *Glossogobius giuris* | India | Choudhary et al., 2023 |
| OR420917 | *P. srivastavai* | *Heteropneustes fossilis* | India |  |
| OP609709 | *P. tripathi* | *Xenentodon cancila* | India | Rajput et al., unpublished |
| MT218558 | *Xystretrum solidum* | *Sphoeroides testudineus* | Mexico | Martínez-Aquino et al., 2020 |
| MT218559 |  |  |  |  |
| De-novo  Assembly* | *P. umblae* | *Salvelinus alpinus* | Norway | Present study |
| L049Hxa* |  | *Coregonus lavaretus* |  |  |
| S043Hxa* |  | *Coregonus lavaretus* |  |  |
| N092Hxb* |  | *Coregonus confusus* | Switzerland |  |
| T005Hxb* |  | *Coregonus steinmanni* |  |  |
| W006Hxb* |  | *Coregonus dúplex* |  |  |
| B110Hxa* |  | *Coregonus fatioi* |  |  |

* **GenBank accession numbers: draft genome: JBPKAA000000000, partial sequences: PV849597-PV849602**

# S3. GENERAL STATISTICS

## GENETIC DIVERSITY

The genetic statistics presented here are based on genotype likelihoods estimated for *P. umblae* using ANGSD (Korneliussen et al., 2014). These statistics provide insights into the genetic diversity and population structure of the parasite across its sampled populations in both subarctic and perialpine lakes.

Table S3. Summary statistics of genetic diversity in *Phyllodistomum umblae* populations from each lake. Columns are the following: the lake; the number of samples; the total number of sites with a depth of coverage of at least 5 in at least 18 samples in Thun and in Langfjordvatn, or in at least ⅘ of the total number of samples in the rest of lakes; S is the number of variable sites identified with SNP p-value threshold of 1.0E-06; H_O_ is the average observed heterozygosity estimated from posterior genotype probabilities obtained with ANGSD; H_E_ is the nucleotide diversity or expected heterozygosity estimated with Tajima’s estimator (Fu, 1994) from the folded site frequency spectrum, which in turn was estimated with the realSFS program from genotype likelihoods obtained with ANGSD (Korneliussen et al., 2014); F is the average inbreeding coefficient estimated per individual with the ngsF program (Vieira et al., 2013), from genotype likelihoods.

| **Lake** | **Samples** | **Total sites** | **S** | **H_O_** | **H_E_** | **F** |
| --- | --- | --- | --- | --- | --- | --- |
| **Perialpine lakes** |  |  |  |  |  |  |
| Bienne | 7 | 165454 | 1293 | 0.0059 | 0.0040 | 0.0002 |
| Brienz | 18 | 116135 | 1359 | 0.0071 | 0.0047 | 0.0000 |
| Thun | 53 | 1001767 | 7331 | 0.0031 | 0.0023 | 0.0002 |
| Walen | 8 | 157706 | 1338 | 0.0062 | 0.0041 | 0.0000 |
| **Subarctic lakes** |  |  |  |  |  |  |
| Langfjordvatn | 27 | 129033 | 2064 | 0.0073 | 0.0052 | 0.0026 |
| Suopatjavri | 20 | 103877 | 1585 | 0.0084 | 0.0057 | 0.0006 |

## REFERENCE GENOME STATISTICS

*Phyllodistomum umblae* draft genome revealed moderate completeness, with 44.6% of the expected genes identified as complete, and a total length of 450 Mb, according to BUSCO (Manni et al., 2021) results. These results are consistent with genome assemblies for non-model flatworms.

Whole draft genome has been deposited at DDBJ/ENA/GenBank under the accession JBPKAA000000000, project number PRJNA1282119.

Table S4. Summary statistics and BUSCO results for the de-novo genome assembly for *Phyllodistomum umblae.*

| BUSCO results (%) |  |
| --- | --- |
| Complete BUSCOs | 44.6 |
| Complete and single-copy BUSCOs | 43.1 |
| Complete and duplicated BUSCOs | 1.5 |
| Fragmented BUSCOs | 9.0 |
| Missing BUSCOs | 46.4 |
| Assembly statistics |  |
| Number of scaffolds | 8,878 |
| Max scaffold length | 520,276 |
| Total length | 450,667,197 |
| Scaffold N50 | 88,476 |

# S4. EXTENDED POPULATION GENETIC ANALYSIS

## PCA BY REGION

To further investigate genetic differentiation within each geographic region, we performed separate PCA analyses for the perialpine and subarctic populations. The PCA of perialpine lakes revealed a large cluster encompassing all sampled populations, with no clear separation among lakes. This suggests no genetic differentiation across perialpine populations, as explained in the manuscript. In contrast, the PCA of subarctic lakes indicated low genetic differentiation, with some distinction between Langfjordvatn and Suohpatjávri populations and between Walen and the other perialpine lakes.


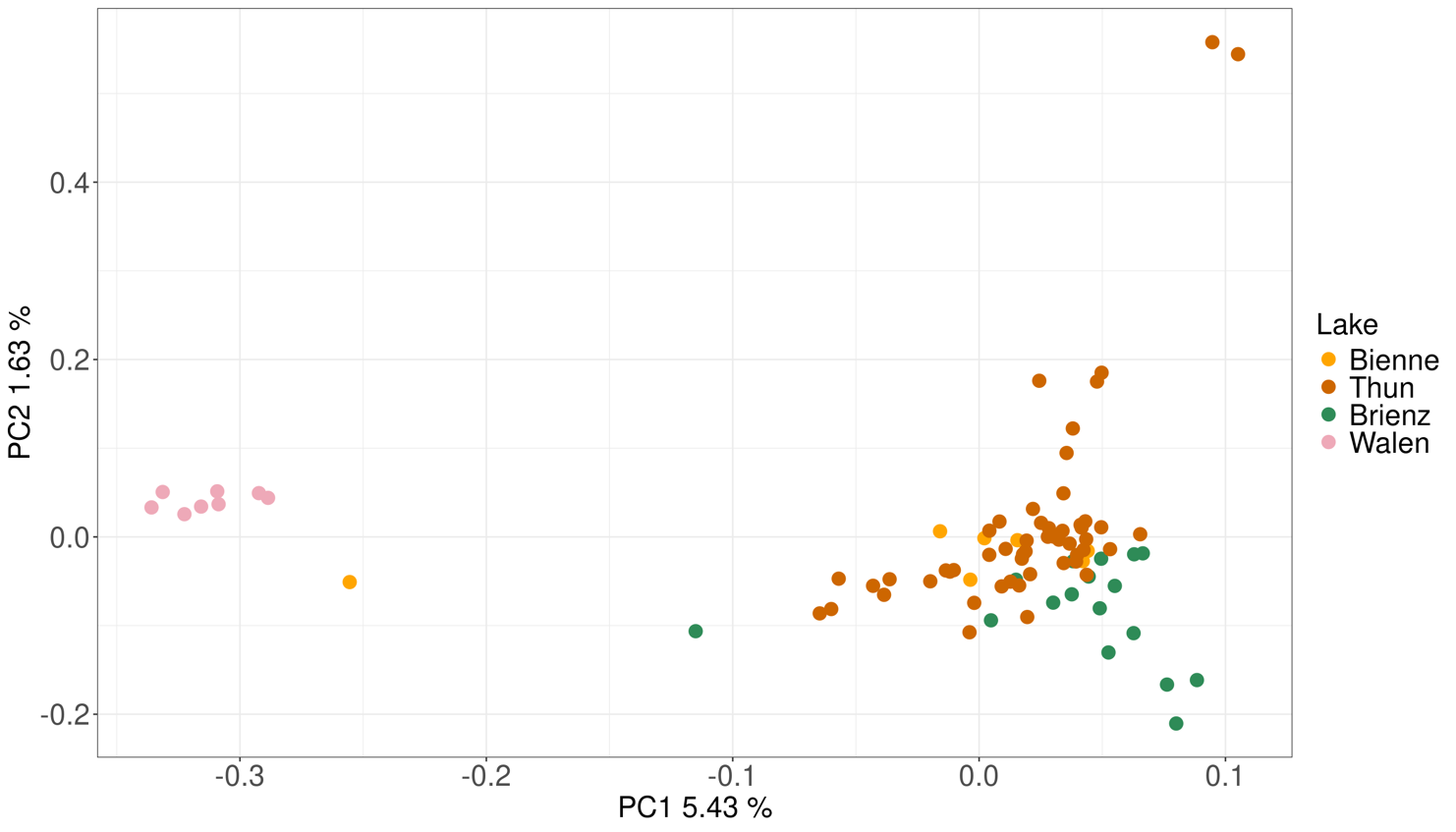


Fig. S3. Principal component analysis across perialpine lakes for *Phyllodistomum umblae*. Each dot represents an individual and the color represents the different lakes. The perialpine populations of *P. umblae* are closely related, with Walen population being the most divergent.


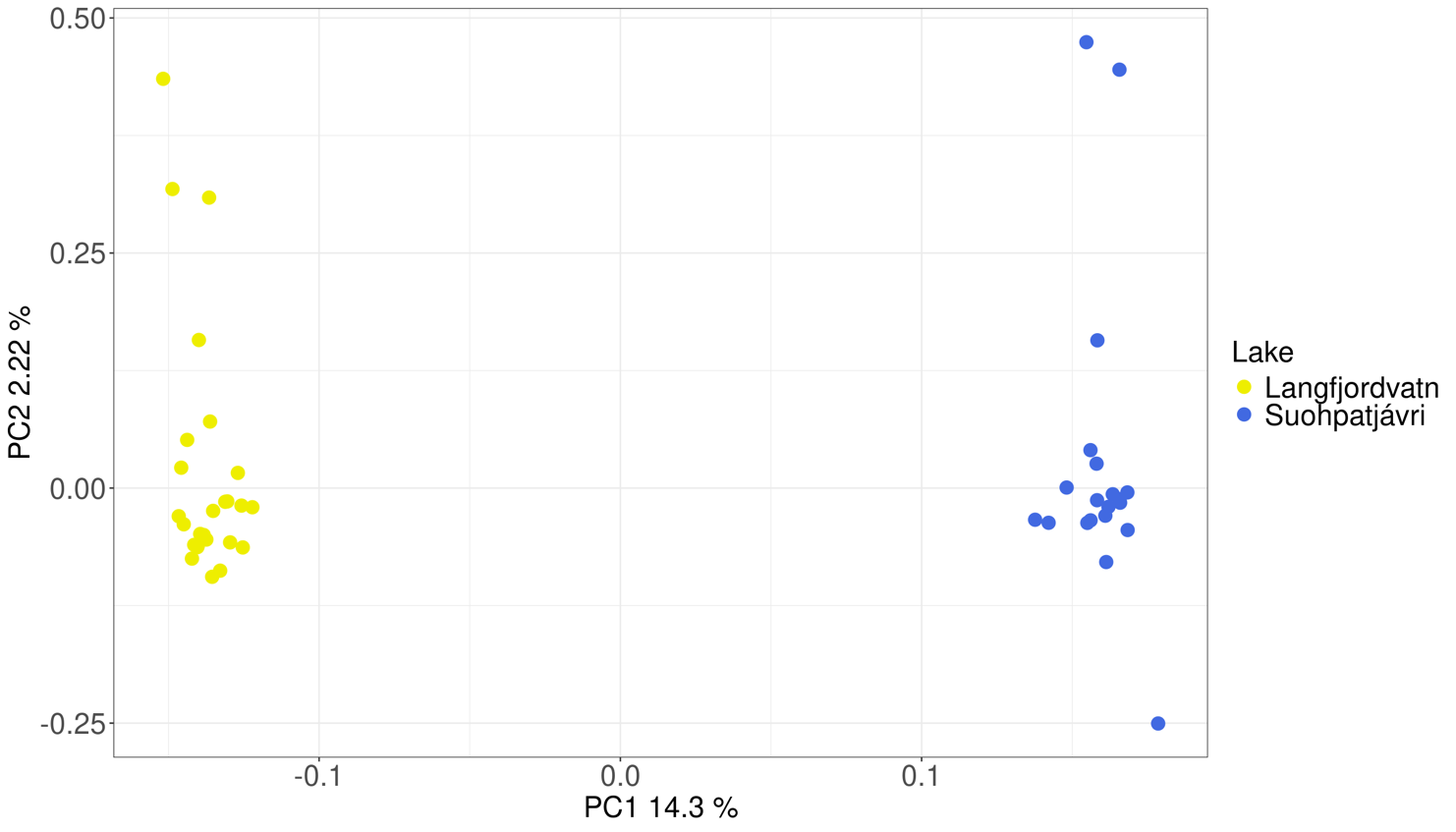


Fig. S4. Principal component analysis across subarctic lakes for *Phyllodistomum umblae*. Each dot represents an individual and the color represents the different lakes. Only the first axis (PC1) differentiate the populations between Langfjordvatn and Suohpatjávri.

## ADMIXTURE BY REGION

We further explored population structure within each region using NGSadmix (Skotte et al., 2013) and EvalAdmix (Garcia-Erill & Albrechtsen, 2020). In the perialpine region, most lake populations exhibit substantial genetic mixing. The exception was Lake Walen, whose individuals formed a slightly differentiated genetic cluster compared to the other perialpine lakes. In the subarctic region, a clear differentiation between Langfjordvatn and Suohpatjávri populations was observed, but evidence of genetic connectivity between these lakes was also detected, reflecting historical or ongoing gene flow.


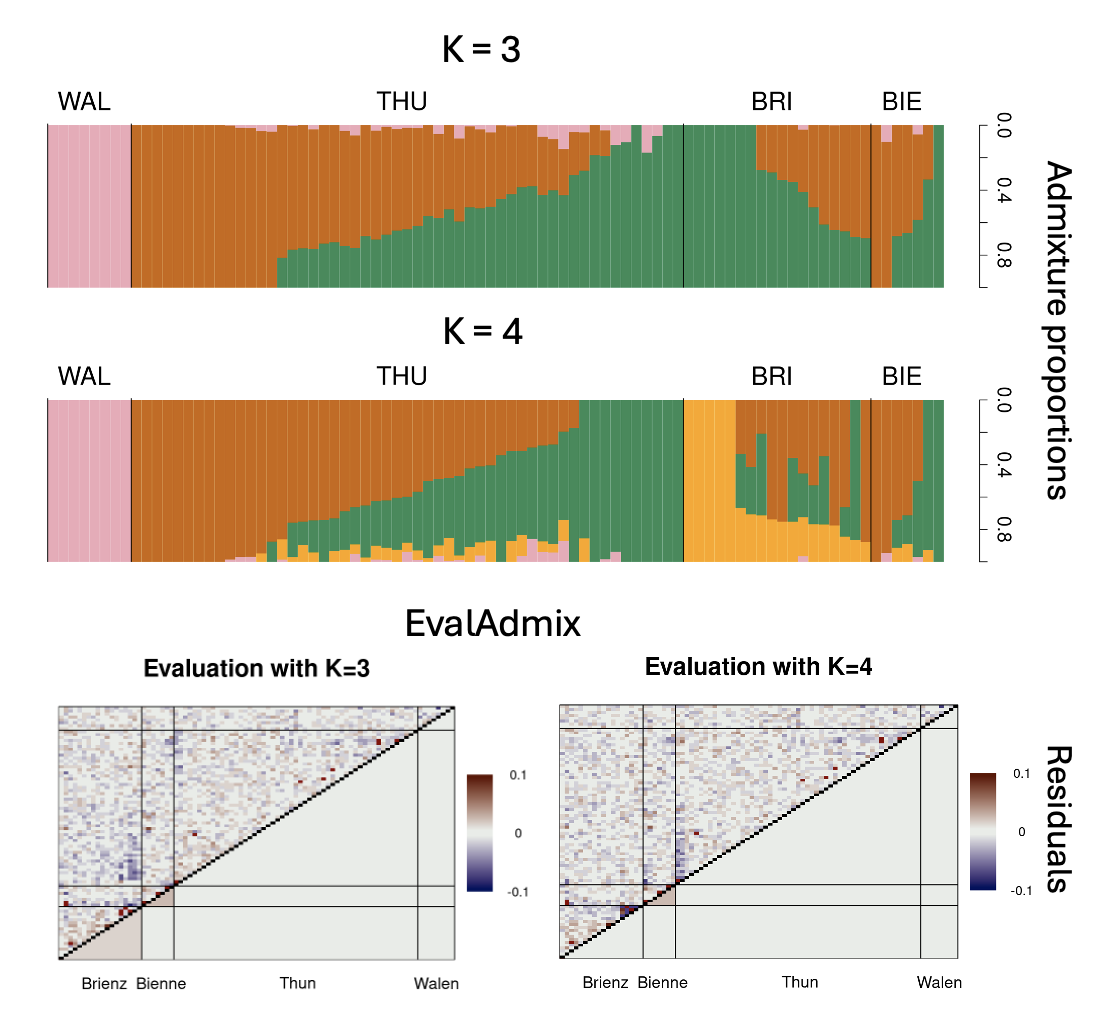


Fig. S5. Ancestry proportions from NGSadmix analysis, where the most adequate number of clusters for perialpine populations of *P. umblae* was K = 4 according to evalAdmix (residuals closest to 0). WAL = Walen, THU = Thun, BRI = Brienz and BIE = Bienne.


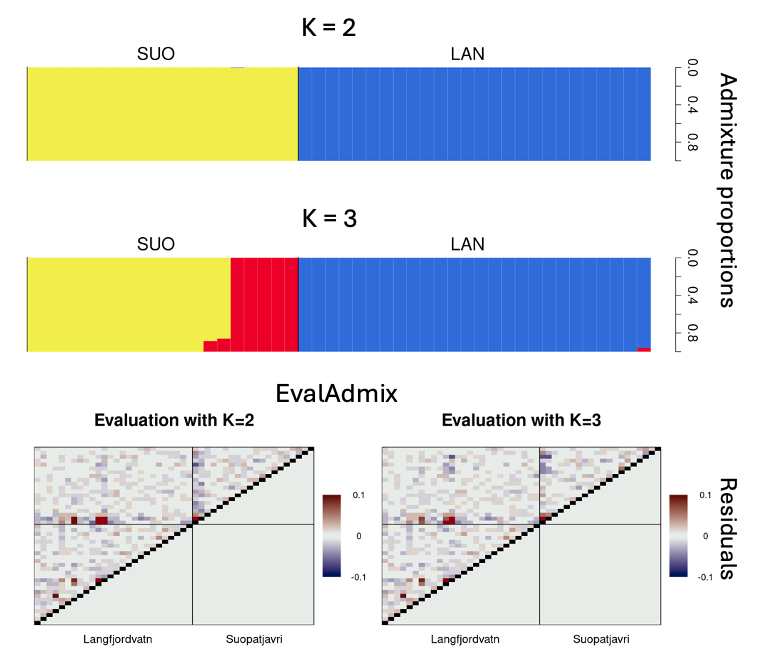


Fig. S6. Ancestry proportions from NGSadmix analysis, where the most adequate number of clusters for subarctic populations of *P. umblae* was K = 2 or K = 3 (both with the same probability) according to evalAdmix. LAN = Langfjordvatn and SUO = Suohpatjávri.

## COMPARISON F_ST_ BETWEEN POPULATIONS

Here, we provide a comparison of the genetic differentiation (F_ST_ values) between populations of *P. umblae* from different lakes. Pairwise F_ST_ values were estimated among these populations using the folded site frequency spectrum (SFS) with realSFS (Korneliussen et al., 2014). The parwise F_ST_ statistic quantifies the proportion of genetic variance that can be attributed to population differences, as opposed to variance within populations. The F_ST_ value ranges from 0 to 1, where 0 indicates no genetic differentiation (i.e., populations are genetically identical) and 1 indicates complete genetic differentiation (i.e., populations are entirely distinct). Negative values are not significant different from zero, and are due to the estimation method. Pairwise F_ST_ values are used to compare the genetic structure of populations in pairs, providing insights into the degree of genetic isolation or connectivity between them. For *P. umblae*, there is moderate differentiation between populations from Suohpatjávri and other lakes, with the highest value between Suohpatjávri and Walen, suggesting a significant but moderate genetic differentiation.

Table S5. Comparisons of *Phyllodistomum umblae* population genetic structure (F_ST_) amongst lakes.

| **Population** | **Population** | **F_ST_ weighted** |
| --- | --- | --- |
|  |  |  |
| Perialpine region | Subarctic region | 0.2568 |
| Bienne | Brienz | -0.0210 |
| Bienne | Langfjordvatn | 0.1676 |
| Brienz | Langfjordvatn | 0.2182 |
| Bienne | Suohpatjávri | 0.1833 |
| Brienz | Suohpatjávri | 0.2272 |
| Langfjordvatn | Suohpatjávri | 0.0088 |
| Bienne | Thun | -0.0154 |
| Brienz | Thun | -0.0093 |
| Langfjordvatn | Thun | 0.2017 |
| Suohpatjávri | Thun | 0.2136 |
| Bienne | Walen | -0.0278 |
| Brienz | Walen | -0.0210 |
| Langfjordvatn | Walen | 0.2228 |
| Suohpatjávri | Walen | 0.2580 |
| Thun | Walen | -0.0102 |

# S5. EXTENDED DEMOGRAPHIC INFERENCE ANALYSIS

## DEMOGRAPHIC INFERENCE BY LAKE POPULATIONS

To evaluate the demographic inference at the lake population level, we generated the folded site frequency spectrum (SFS) for each *P. umblae* and *Pr. fallx* populations. However, the SFS distribution of *P. umblae* lake populations exhibited a strong bias towards singletons in some populations, which could be caused by sequencing errors at low-coverage sites, and casts doubts on the adequacy of the data for this kind of inference. In addition, the relatively small sample sizes per lake further constrained the inference of lake-level demographic histories. Given that demographic inference relies on the shape of the SFS to reconstruct past population size changes, the combination of low sample sizes and skewed allele frequency distributions rendered demographic reconstructions at the lake level unreliable for *P. umblae*. On the other hand, for *Pr. fallax*, the SFS distributions were more balanced, and the number of samples per population was considerably larger, allowing for demographic inference at the lake population level.


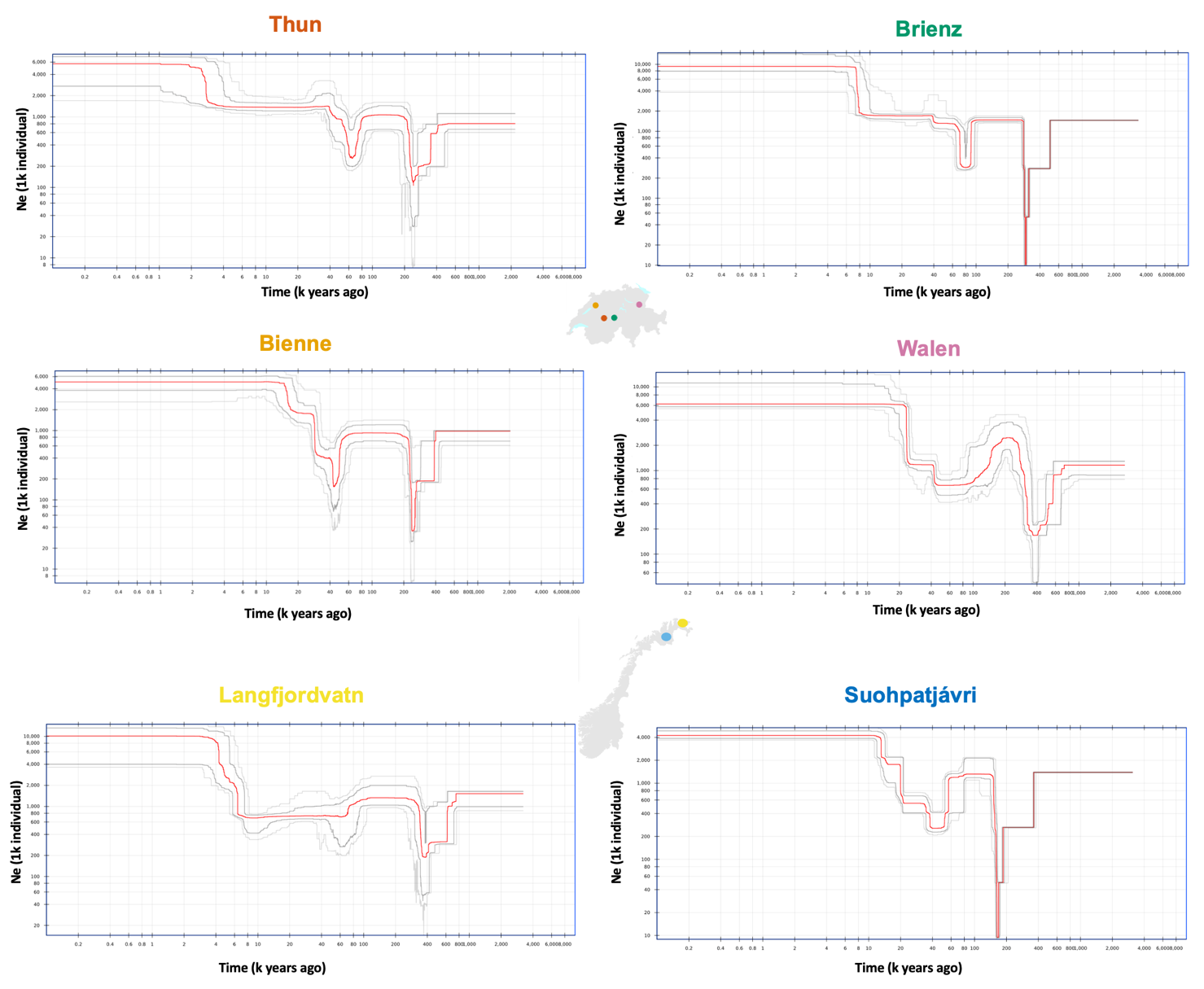


Fig. S7. Demographic inference for each population (i.e., lake) in perialpine and subarctic region of *Pr. fallax*.

## ESTIMATION OF EVOLUTIONARY RATE

To estimate the evolutionary mutation rate per nucleotide per year for both *P. umblae* and *Pr. fallax*, we applied a regression-based approach following Lynch (2010). This method relies on the observed relationship between log-transformed genome size and log-transformed mutation rates across a wide range of taxa. We compiled empirical mutation rate estimates from published genomic studies, including those of *Taenia* species (Wang et al., 2016) and *Ligula intestinalis* (Nazarizadeh et al., 2023), in addition to the values reported in Lynch (2010).

A linear regression model was fitted to these data (Fig. S8), with genome size as the independent variable and mutation rate as the dependent variable. The resulting regression equation was used to infer mutation rates for *P. umbla*e and *Pr. fallax* based on their estimated genome sizes. The model yielded mutation rate estimates of 3.66 × 10⁻⁹ substitutions/site/year for *P. umblae* and 5.06 × 10⁻⁹ substitutions/site/year for *Pr. fallax*, used in the Stairway Plot demographic inference analysis.

The choice of this method is justified by its ability to provide species-specific estimates based on empirical genomic data, accounting for the well-documented scaling relationship between genome size and mutation rate. Given the absence of direct experimental mutation rate estimates for these taxa, this regression-based approach represents the most robust alternative for demographic modeling.

*Pr. fallax*

*P. umblae*


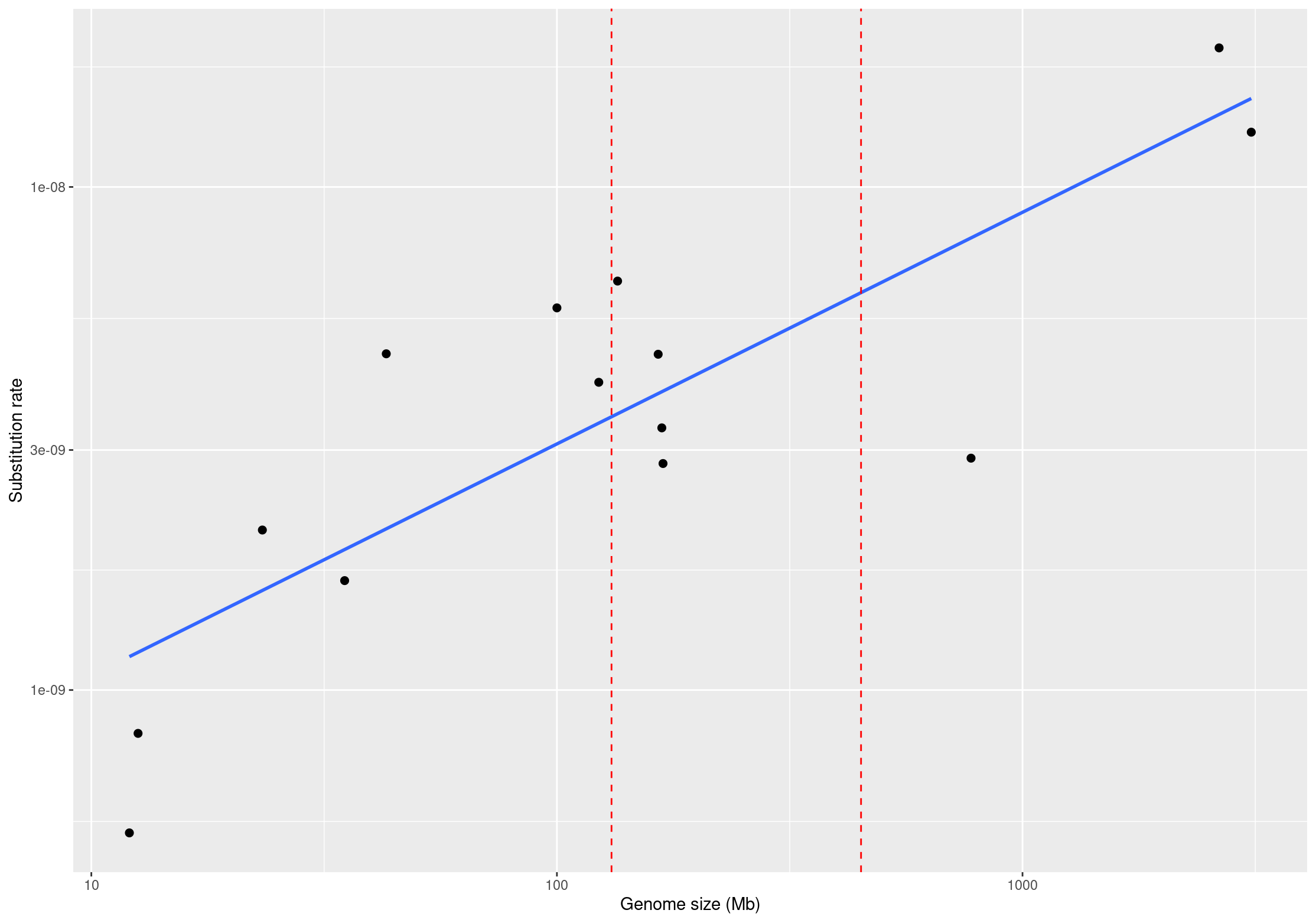


Fig. S8. Estimation of base substitution rate per nucleotide site per generation with genome size. Each data point represents the average estimate for a separate taxon, and the two vertical lines represents the genome sizes for *P. umblae* (450 Mb) and *Pr. fallax* (136 Mb), as obtained from their respective assemblies.

# REFERENCES

Al-Ghamdi, A., Morsy, K., Dajem, S., Shati, A., Al-Kahtani, M., Baiomy, A., & Ezzat, A. (2021). Molecular evidence and morphological aspects of Transversotrema licinum, Phyllodistomum hoggettae, and re-description of Gyliauchen volubilis (Digenea) from the Red Sea. *Veterinary Research Forum*, *12*(1), 15-24. https://doi.org/10.30466/vrf.2018.95586.2296

Altschul, S. F., Gish, W., Miller, W., Myers, E. W., & Lipman, D. J. (1990). Basic local alignment search tool. *Journal of Molecular Biology*, *215*(3), 403-410. https://doi.org/10.1016/S0022-2836(05)80360-2

Camacho, C., Coulouris, G., Avagyan, V., Ma, N., Papadopoulos, J., Bealer, K., & Madden, T. L. (2009). BLAST+: Architecture and applications. *BMC Bioinformatics*, *10*(1), 421. https://doi.org/10.1186/1471-2105-10-421

Castresana, J. (2000). Selection of Conserved Blocks from Multiple Alignments for Their Use in Phylogenetic Analysis. *Molecular Biology and Evolution*, *17*(4), 540-552. https://doi.org/10.1093/oxfordjournals.molbev.a026334

Choudhary, K., Ray, S., Agrawal, N., & Shamsi, S. (2023). Genetic characterization and phylogenetic relationships of Phyllodistomum parasites in Indian subcontinent: Insights from freshwater fish and shrimp hosts. *Parasitology Research*, *122*(10), 2301-2315. https://doi.org/10.1007/s00436-023-07930-3

Cutmore, S. C., & Cribb, T. H. (2018). Two species of Phyllodistomum Braun, 1899 (Trematoda: Gorgoderidae) from Moreton Bay, Australia. *Systematic Parasitology*, *95*(4), 325-336. https://doi.org/10.1007/s11230-018-9784-2

Cutmore, S. C., Miller, T. L., Curran, S. S., Bennett, M. B., & Cribb, T. H. (2013). Phylogenetic relationships of the Gorgoderidae (Platyhelminthes: Trematoda), including the proposal of a new subfamily (Degeneriinae n. subfam.). *Parasitology Research*, *112*(8), 3063-3074. https://doi.org/10.1007/s00436-013-3481-5

Faltýnková, A., Pantoja, C., Skírnisson, K., & Kudlai, O. (2020). Unexpected diversity in northern Europe: Trematodes from salmonid fishes in Iceland with two new species of Crepidostomum Braun, 1900. *Parasitology Research*, *119*(8), 2439-2462. https://doi.org/10.1007/s00436-020-06724-1

Fu, Y. X. (1994). Estimating effective population size or mutation rate using the frequencies of mutations of various classes in a sample of DNA sequences. *Genetics*, *138*(4), 1375-1386. https://doi.org/10.1093/genetics/138.4.1375

Garcia-Erill, G., & Albrechtsen, A. (2020). Evaluation of model fit of inferred admixture proportions. *Molecular Ecology Resources*, *20*(4), 936-949. https://doi.org/10.1111/1755-0998.13171

Gosho, M., Itsukushima, S., Collins, M. H., Dalton, I. T., Rosen, R. B., & Urabe, M. (2023). Molecular phylogenetic position and the life cycle of Phyllodistomum cyprini Feng et Wang, 1995 in Japan, with a note of a larval Phyllodistomum sp. From unionid mussels in North America. *Parasitology International*, 102807. https://doi.org/10.1016/j.parint.2023.102807

Katoh, K., Misawa, K., Kuma, K., & Miyata, T. (2002). MAFFT: A novel method for rapid multiple sequence alignment based on fast Fourier transform. *Nucleic Acids Research*, *30*(14), 3059-3066. https://doi.org/10.1093/nar/gkf436

Korneliussen, T. S., Albrechtsen, A., & Nielsen, R. (2014). ANGSD: Analysis of Next Generation Sequencing Data. *BMC Bioinformatics*, *15*(1), 356. https://doi.org/10.1186/s12859-014-0356-4

Kozlov, A. M., Darriba, D., Flouri, T., Morel, B., & Stamatakis, A. (2019). RAxML-NG: A fast, scalable and user-friendly tool for maximum likelihood phylogenetic inference. *Bioinformatics*, *35*(21), 4453-4455. https://doi.org/10.1093/bioinformatics/btz305

León, G. P.-P. D., Martínez-Aquino, A., & Mendoza-Garfias, B. (2015). Two new species of Phyllodistomum Braun, 1899 (Digenea: Gorgoderidae), from freshwater fishes (Cyprinodontiformes: Goodeidae: Goodeinae) in central Mexico: An integrative taxonomy approach using morphology, ultrastructure and molecular phylogenetics. *Zootaxa*, *4013*(1), Article 1. https://doi.org/10.11646/zootaxa.4013.1.6

León, G. P.-P. de, Pinacho-Pinacho, C. D., Mendoza-Garfias, B., & García-Varela, M. (2015). Phyllodistomum spinopapillatum sp. nov. (Digenea: Gorgoderidae), from the Oaxaca killifish Profundulus balsanus (Osteichthyes: Profundulidae) in Mexico, with new host and locality records of P. inecoli: Morphology, ultrastructure and molecular evidence. *Acta Parasitologica*, *60*(2), 298-307. https://doi.org/10.1515/ap-2015-0042

Li, H., Handsaker, B., Wysoker, A., Fennell, T., Ruan, J., Homer, N., Marth, G., Abecasis, G., Durbin, R., & 1000 Genome Project Data Processing Subgroup. (2009). The Sequence Alignment/Map format and SAMtools. *Bioinformatics*, *25*(16), 2078-2079. https://doi.org/10.1093/bioinformatics/btp352

Lynch, M. (2010). Evolution of the mutation rate. *Trends in Genetics*, *26*(8), 345-352. https://doi.org/10.1016/j.tig.2010.05.003

Manni, M., Berkeley, M. R., Seppey, M., & Zdobnov, E. M. (2021). BUSCO: Assessing Genomic Data Quality and Beyond. *Current Protocols*, *1*(12), e323. https://doi.org/10.1002/cpz1.323

Martínez-Aquino, A., García-Teh, J. G., Ceccarelli, F. S., Aguilar-Aguilar, R., Vidal-Martinez, V. M., & Aguirre-Macedo, M. L. (2020). New morphological and molecular data for Xystretrum solidum (Gorgoderidae, Gorgoderinae) from Sphoeroides testudineus (Tetraodontiformes, Tetraodontidae) in Mexican waters. *ZooKeys*, *925*, 141-161. https://doi.org/10.3897/zookeys.925.49503

Nazarizadeh, M., Nováková, M., Loot, G., Gabagambi, N. P., Fatemizadeh, F., Osano, O., Presswell, B., Poulin, R., Vitál, Z., Scholz, T., Halajian, A., Trucchi, E., Kočová, P., & Štefka, J. (2023). Historical dispersal and host-switching formed the evolutionary history of a globally distributed multi-host parasite – The *Ligula intestinalis* species complex. *Molecular Phylogenetics and Evolution*, *180*, 107677. https://doi.org/10.1016/j.ympev.2022.107677

Olson, P. D., Cribb, T. H., Tkach, V. V., Bray, R. A., & Littlewood, D. T. J. (2003). Phylogeny and classification of the Digenea (Platyhelminthes: Trematoda). *International Journal for Parasitology*, *33*(7), 733-755. https://doi.org/10.1016/s0020-7519(03)00049-3

Paradis, E., & Schliep, K. (2019). ape 5.0: An environment for modern phylogenetics and evolutionary analyses in R. *Bioinformatics*, *35*(3), 526-528. https://doi.org/10.1093/bioinformatics/bty633

Petkevičiūtė, R., Kudlai, O., Stunžėnas, V., & Stanevičiūtė, G. (2015). Molecular and karyological identification and morphological description of cystocercous cercariae of Phyllodistomum umblae and Phyllodistomum folium (Digenea, Gorgoderidae) developing in European sphaeriid bivalves. *Parasitology International*, *64*(5), 441-447. https://doi.org/10.1016/j.parint.2015.06.007

Petkevičiūtė, R., Stunžėnas, V., & Stanevičiūtė, G. (2023). Hidden Diversity in European Allocreadium spp. (Trematoda, Allocreadiidae) and the Discovery of the Adult Stage of Cercariaeum crassum Wesenberg-Lund, 1934. *Diversity*, *15*(5), Article 5. https://doi.org/10.3390/d15050645

Petkevičiūtė, R., Stunžėnas, V., Stanevičiūtė, G., & Zhokhov, A. E. (2015). European Phyllodistomum (Digenea, Gorgoderidae) and phylogenetic affinities of Cercaria duplicata based on rDNA and karyotypes. *Zoologica Scripta*, *44*(2), 191-202. https://doi.org/10.1111/zsc.12080

Petkevičiūtė, R., Zhokhov, A. E., Stunžėnas, V., Poddubnaya, L. G., & Stanevičiūtė, G. (2020). Phyllodistomum kupermani n. sp. from the European perch, Perca fluviatilis L. (Perciformes: Percidae), and redescription of Phyllodistomum macrocotyle (Lühe, 1909) with notes on the species diversity and host specificity in the European Phyllodistomum spp. (Trematoda: Gorgoderidae). *Parasites & Vectors*, *13*(1), 561. https://doi.org/10.1186/s13071-020-04434-2

Pinacho-Pinacho, C. D., Sereno-Uribe, A. L., Hernández-Orts, J. S., García-Varela, M., & León, G. P.-P. de. (2021). Integrative taxonomy reveals an even greater diversity within the speciose genus Phyllodistomum (Platyhelminthes: Trematoda : Gorgoderidae), parasitic in the urinary bladder of Middle American freshwater fishes, with descriptions of five new species. *Invertebrate Systematics*, *35*(7), 754-775. https://doi.org/10.1071/IS21007

R Core Team (2024). R: A Language and Environment for Statistical Computing. R Foundation for Statistical Computing, Vienna, Austria. https://www.R-project.org/.

Razo-Mendivil, U., Pérez-Ponce de León, G., & Rubio-Godoy, M. (2013). Integrative taxonomy identifies a new species of Phyllodistomum (Digenea: Gorgoderidae) from the twospot livebearer, Heterandria bimaculata (Teleostei: Poeciliidae), in Central Veracruz, Mexico. *Parasitology Research*, *112*(12), 4137-4150. https://doi.org/10.1007/s00436-013-3605-y

Rosas-Valdez, R., Choudhury, A., & De León, G. P.-P. (2011). Molecular prospecting for cryptic species in Phyllodistomum lacustri (Platyhelminthes, Gorgoderidae). *Zoologica Scripta*, *40*(3), 296-305. https://doi.org/10.1111/j.1463-6409.2011.00472.x

Skotte, L., Korneliussen, T. S., & Albrechtsen, A. (2013). Estimating Individual Admixture Proportions from Next Generation Sequencing Data. *Genetics*, *195*(3), 693-702. https://doi.org/10.1534/genetics.113.154138

Stunžėnas, V., Petkevičiūtė, R., Poddubnaya, L. G., Stanevičiūtė, G., & Zhokhov, A. E. (2017). Host specificity, molecular phylogeny and morphological differences of Phyllodistomum pseudofolium Nybelin, 1926 and Phyllodistomum angulatum Linstow, 1907 (Trematoda: Gorgoderidae) with notes on Eurasian ruffe as final host for Phyllodistomum spp. *Parasites & Vectors*, *10*(1), 286. https://doi.org/10.1186/s13071-017-2210-9

Urabe, M., Ishibashi, R., & Uehara, K. (2015). The life cycle and molecular phylogeny of a gorgoderid trematode recorded from the mussel Nodularia douglasiae in the Yodo River, Japan. *Parasitology International*, *64*(1), 26-32. https://doi.org/10.1016/j.parint.2014.09.003

Vasimuddin, Md., Misra, S., Li, H., & Aluru, S. (2019). Efficient Architecture-Aware Acceleration of BWA-MEM for Multicore Systems. *2019 IEEE International Parallel and Distributed Processing Symposium (IPDPS)*, 314-324. https://doi.org/10.1109/IPDPS.2019.00041

Vieira, F. G., Fumagalli, M., Albrechtsen, A., & Nielsen, R. (2013). Estimating inbreeding coefficients from NGS data: Impact on genotype calling and allele frequency estimation. *Genome Research*, *23*(11), 1852-1861. https://doi.org/10.1101/gr.157388.113

Wang, S., Wang, S., Luo, Y., Xiao, L., Luo, X., Gao, S., Dou, Y., Zhang, H., Guo, A., Meng, Q., Hou, J., Zhang, B., Zhang, S., Yang, M., Meng, X., Mei, H., Li, H., He, Z., Zhu, X., … Cai, X. (2016). Comparative genomics reveals adaptive evolution of Asian tapeworm in switching to a new intermediate host. *Nature Communications*, *7*. https://doi.org/10.1038/ncomms12845
